# ICAM2 promotes endocrine resistance via dynein-mediated OXPHOS activation in ER-positive breast cancer

**DOI:** 10.1038/s41419-026-08864-1

**Published:** 2026-05-18

**Authors:** Si Chen, Shiyi Wu, Jiajie Hu, Bohan Liu, Yuting Liu, Siyue Yang, Fen Tang, Yiqing He, Qinqing Liu, Yiwen Liu, Yan Du, Guoliang Zhang, Qian Guo, Feng Gao, Cuixia Yang

**Affiliations:** 1 Department of Clinical Laboratory & Department of Molecular Biology, Shanghai Sixth People’s Hospital Affiliated to Shanghai Jiao Tong University School of Medicine, Shanghai, China; 2 Medical Laboratory Science, College of Health Science and Technology, Shanghai Jiao Tong University School of Medicine, Shanghai, China; 3 Department of Breast Surgery, Shanghai Sixth People’s Hospital Affiliated to Shanghai Jiao Tong University School of Medicine, Shanghai, China

**Keywords:** Breast cancer, Membrane proteins

## Abstract

Acquired endocrine resistance in ER^+^ breast cancer (BC) involves metabolic reprogramming, yet key drivers are unclear. Multi-omics of endocrine-resistant BC revealed upregulated oxidative phosphorylation (OXPHOS) and identified intercellular adhesion molecule 2 (ICAM2) as a biomarker of high-OXPHOS cells. ICAM2-positive cells were significantly enriched in resistant tumors and predicted poor survival, and were functionally essential for maintaining resistance and promoting metastasis in vivo. Mechanistically, ICAM2 binds dynein light chain DYNLT3 and the mitochondrial complex I subunit MT-ND2, thereby facilitating dynein-mediated mitochondrial trafficking and further modulating the assembly of mitochondrial complex I. Disrupting this interaction through ICAM2 knockdown or dynein inhibition (Ciliobrevin D) effectively suppressed OXPHOS activity. Importantly, ERα inhibition alleviates the transcriptional repression of ICAM2 by ERα. Therapeutically, combining the complex I inhibitor IACS-10759 with fulvestrant potently inhibited both tumor growth and metastasis. Collectively, these findings reveal that ICAM2 drives endocrine resistance via dynein-dependent OXPHOS activation, revealing a targetable axis in refractory ER^+^ BC.

**Figure:**
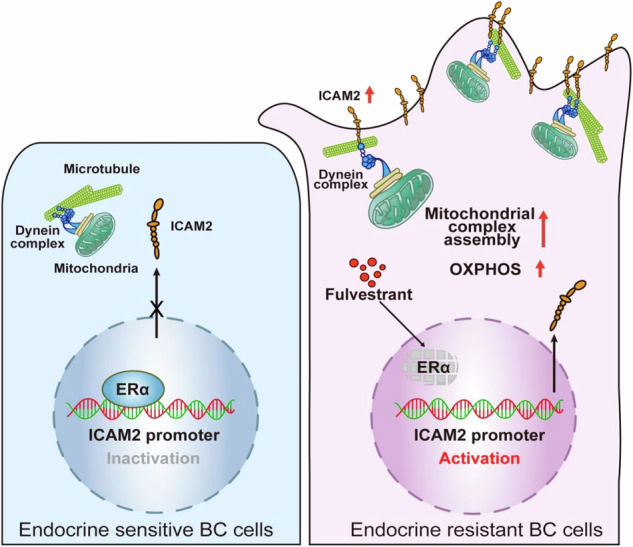
In summary, we establish ICAM2 as a novel biomarker and driver of endocrine resistance in ER⁺ breast cancer. ICAM2⁺ cancer cells—enriched in treatment-resistant tumors—maintain elevated OXPHOS by assembling a functional complex with dynein and mitochondrial Complex I, thereby promoting mitochondrial trafficking. Disruption of this axis, either through ICAM2 depletion or Complex I inhibition, re-sensitizes tumors to therapy, revealing a targetable metabolic dependency in resistant disease.

In summary, we establish ICAM2 as a novel biomarker and driver of endocrine resistance in ER⁺ breast cancer. ICAM2⁺ cancer cells—enriched in treatment-resistant tumors—maintain elevated OXPHOS by assembling a functional complex with dynein and mitochondrial Complex I, thereby promoting mitochondrial trafficking. Disruption of this axis, either through ICAM2 depletion or Complex I inhibition, re-sensitizes tumors to therapy, revealing a targetable metabolic dependency in resistant disease.

## Introduction

Breast cancer (BC) is the most common malignancy in women globally, responsible for approximately 32% of new cases and 14% of cancer-related deaths among female patients [1]. The estrogen receptor-positive (ER^+^) subtype represents about 70% of all BCs [2]. Endocrine therapy—which includes selective ER modulators (e.g., tamoxifen), selective ER degraders (e.g., fulvestrant), and aromatase inhibitors (e.g., anastrozole)—serves as the cornerstone treatment for ER^+^ disease [3]. However, acquired resistance develops in over 30% of patients, ultimately limiting therapeutic success [4, 5]. While several resistance mechanisms—such as ER mutations and activation of alternative signaling pathways—have been described, clinically applicable biomarkers for predicting resistance and effective salvage strategies remain an unmet need.

Mitochondrial metabolic reprogramming, with oxidative phosphorylation (OXPHOS) at its core, is a pivotal mechanism underlying cancer drug resistance [6, 7]. OXPHOS supports tumors by generating energy, remodeling the microenvironment, and facilitating biosynthesis. Oncogenic pathway activation-driven enhancement of OXPHOS provides a survival advantage, enabling adaptation and resistance to chemotherapy, targeted therapy, and immunotherapy [8–11]. This is evidenced by OXPHOS-dependent subpopulations in chemoresistant BC [12–15], OXPHOS-reprogrammed immunosuppressive neutrophils [16], and its role in endocrine therapy resistance [17–19]. Consequently, targeting OXPHOS and exploiting mitochondrial vulnerabilities offer a strategic avenue to overcome therapy resistance. Preclinically, OXPHOS inhibition alleviates hypoxia—by reducing oxygen consumption—to potentiate radio- and immunotherapy [20]. However, this benefit is likely restricted to patients with consumption-driven hypoxia, and its clinical translation remains unproven. Moreover, such inhibition fails to counteract, and may even exacerbate, glycolytic acidosis and its associated immunosuppression. Crucially, how cancer cells activate oncogenic pathways to enhance OXPHOS and induce mitochondrial adaptations in a context-dependent way that confers a survival advantage remains unclear.

Intercellular adhesion molecule-2 (ICAM2) is a transmembrane glycoprotein that promotes lymphocyte recirculation, vascular permeability, and breast cancer metastasis [21–23]. Yet, its functional role in driving therapy resistance remains elusive. In this study, ICAM2 is identified as a master regulator of OXPHOS in ER^+^ BC during acquired endocrine resistance. Multi-omics analysis of fulvestrant-resistant ER^+^ tumors reveals a coordinated metabolic shift toward robust OXPHOS activation, accompanied by suppressed glycolysis. Single-cell RNA sequencing identifies ICAM2 as a specific marker of high-OXPHOS cells within resistant populations, with its expression correlating with enhanced mitochondrial function, metastatic progression, and poor patient survival. Mechanistically, ICAM2 forms a functional complex with the dynein light chain DYNLT3 and the mitochondrial complex I subunit MT-ND2, facilitating mitochondrial trafficking and respirasome assembly to sustain OXPHOS. ERα transcriptionally represses ICAM2, and its loss upon endocrine therapy derepresses ICAM2, driving the OXPHOS-high phenotype. Accordingly, combining the complex I inhibitor IACS-10759 with fulvestrant potently suppresses tumor growth and metastasis in vivo, validating the therapeutic relevance of this axis.

This study establishes ICAM2 as a central node linking mitochondrial metabolism to endocrine resistance, providing a prognostic biomarker for early detection of resistance and a therapeutic strategy to resensitize resistant tumors by dual targeting of ERα and mitochondrial metabolism.

## Results

### Multi-omics identifies enhanced mitochondrial OXPHOS in acquired endocrine resistance

**Fig. 1:**
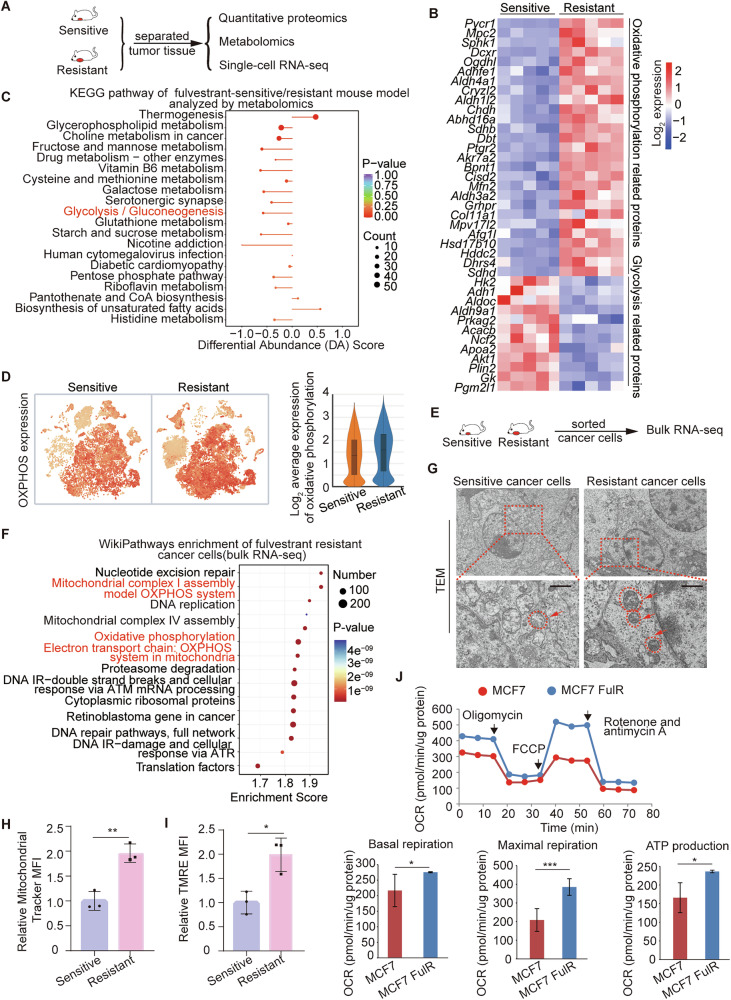
Mitochondrial remodeling underlies fulvestrant resistance in ER^+^ breast cancer models. **A** Schematic diagram of a fulvestrant-resistant ER^+^ breast cancer mouse model for scRNA sequencing, quantitative proteomic profiling, and metabolomics. **B** The heatmap shows relative levels of proteins stratified by quantitative proteomics of tumor tissues derived from a fulvestrant-sensitive/resistant mouse model. **C** KEGG analysis of the differential abundance (DA) score of metabolomics. **D** UMAP plot displaying OXPHOS-related gene sets expression in the fulvestrant-sensitive and -resistant mouse model. **E** Schematic diagram of fulvestrant-resistant cancer cells for bulk RNA-seq. **F** WikiPathways enrichment analysis of the bulk RNA-seq dataset in resistant cancer cells. **G** Transmission electron microscope (TEM) images showing mitochondria of cancer cells sorted from a fulvestrant-sensitive/resistant mouse model. Red arrows indicate mitochondria. Scale bars, 10 μm. MitoTracker Green staining (mitochondrial mass) (**H**) and TMRE staining (mitochondrial membrane potential) (**I**) of cancer cells isolated from fulvestrant-sensitive/resistant mouse models by FACS. Relative median fluorescence intensity (MFI) shown (*n* = 3 biological replicates per group). **J** Analysis of oxidative capacity of MCF7 and MCF7 FulR cells. The oxygen consumption rate (OCR) is shown following sequential additions of different compounds (oligomycin, FCCP, and rotenone/antimycin A). Basal respiration, ATP production, and maximal respiration are shown individually. *n* = 5 for each group. All data are shown as mean ± SEM. *p < 0.05, **p < 0.01, ***p < 0.001.

Based on a fulvestrant-resistant ER^+^ BC mouse model generated as previously reported [24], we performed integrated proteomic, metabolomic, and single-cell RNA-seq profiling of resistant tumors (Fig. 1A, S1A–C). These analyses revealed a consistent metabolic reprogramming in resistant tumors, characterized by enhanced oxidative phosphorylation (OXPHOS) and reduced glycolysis (Fig. 1B, C). scRNA-seq further confirmed that OXPHOS-related gene sets were specifically upregulated in resistant cancer cells (Fig. 1D; Table S1). To validate this transcriptional signature, we isolated endocrine-sensitive and -resistant cancer cells for bulk RNA-seq (Fig. 1E, S1D). Enrichment analysis of differentially expressed genes (DEGs) demonstrated significant upregulation of mitochondrial OXPHOS pathways—including “Mitochondrial Complex I assembly”, “Oxidative phosphorylation”, and the electron transport chain—in resistant cancer cells (Fig. 1F). Collectively, these multi-omics findings indicate that a marked upregulation of OXPHOS in cancer cells is associated with endocrine resistance, underscoring metabolic rewiring as a key adaptive mechanism in BC.

To validate the multi-omics evidence of metabolic rewiring, we first examined mitochondrial ultrastructure via transmission electron microscopy (TEM). Resistant cancer cells exhibited increased mitochondrial number and more densely packed cristae (Fig. 1G), morphological hallmarks consistent with enhanced oxidative phosphorylation (OXPHOS). This phenotype was further recapitulated in fulvestrant-resistant MCF7 cells (MCF7 FulR) generated in vitro (Fig. S1E). Furthermore, flow cytometry and confocal imaging confirmed a significant increase in mitochondrial mass (Mitochondrial tracker; Fig. 1H, S1F, G) and elevated mitochondrial membrane potential (TMRE; Fig. 1I, S1H, I) in endocrine-resistant cells, both in primary tumors and in vitro models (fulvestrant- or tamoxifen-resistant MCF7). These findings support reinforced mitochondrial OXPHOS activity. Critically, mitochondrial stress tests in MCF7 FulR cells demonstrated markedly elevated basal respiration, maximal respiratory capacity, and ATP production compared to parental cells (Fig. 1J), directly confirming enhanced oxidative respiration. Collectively, these structural, biochemical, and functional results demonstrate that endocrine resistance is mechanistically underpinned by enhanced mitochondrial OXPHOS, thereby independently validating the multi-omics findings.

### Enhanced OXPHOS is associated with ICAM2 upregulation in endocrine-resistant BC

**Fig. 2:**
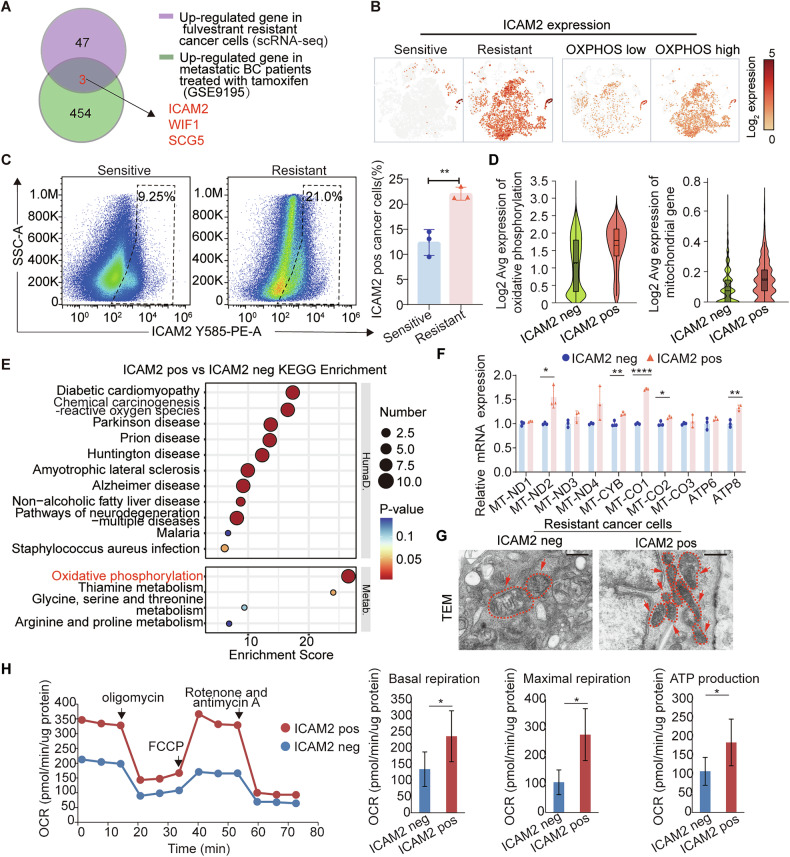
ScRNA-seq identifies ICAM2 as an OXPHOS-high biomarker in endocrine resistance. **A** Overlapping genes upregulated by metastatic breast cancer patients treated with tamoxifen (GSE9195), fulvestrant-resistant cancer cells identified by scRNA-seq. **B** UMAP plot displaying ICAM2 expression in the fulvestrant-sensitive and -resistant mouse model across different cell types (left), and within fulvestrant-resistant tumor cells categorized into OXPHOS low and high group (right). **C** FACS analysis showing the proportion of ICAM2-positive cancer cells in tumors from fulvestrant-sensitive and -resistant mouse models (*n* = 3 per group). **D** Violin plots depict the average expression levels of oxidative phosphorylation and mitochondrial genes between ICAM2-positive and ICAM2-negative fulvestrant-resistant cancer cells. **E** KEGG pathway enrichment analysis of genes upregulated in ICAM2-positive cancer cells sorted from fulvestrant-resistant mouse models by RNA-seq. **F** Relative mRNA level of OXPHOS-related genes in ICAM2-positive and negative cells sorted from fulvestrant-resistant cancer cells. Bars represent mean ± SEM of three independent samples. **G** TEM images showing mitochondria in ICAM2-negative (ICAM2 neg) and -positive (ICAM2 pos) cancer cells sorted from a fulvestrant-resistant mouse model. Red arrows indicate mitochondria. Scale bar, 500 nm. **H** Analysis of the oxidative capacity of ICAM2-positive and negative cells sorted from MCF7 FulR cells. The oxygen consumption rate (OCR) is shown following sequential additions of different compounds (oligomycin, FCCP, and rotenone/antimycin A). Basal respiration, ATP production, and maximal respiration are shown individually. *n* = 5 to 6 for each group. All data are shown as mean ± SEM. *p < 0.05.

To investigate mechanisms of elevated OXPHOS in endocrine resistance, we integrated upregulated genes from tamoxifen-treated metastatic ER^+^ BCs (GSE9195) and from our scRNA-seq data of resistant BC cells. This cross-dataset analysis revealed three candidate genes linked to OXPHOS: *ICAM2*, *WIF1*, and *SCG5* (Fig. 2A). We then stratified BC cells into OXPHOS-low and OXPHOS-high groups based on median OXPHOS gene set expression (Table S1). UMAP visualization indicated that, among the candidates, ICAM2 exhibited the most pronounced upregulation in resistant BC cells and displayed the strongest spatial association with the high OXPHOS state (Fig. 2B, S2A).

To determine whether ICAM2 is linked to OXPHOS and endocrine resistance, we first quantified its expression in primary cancer cells from resistant tumors. Flow cytometry revealed a significantly higher proportion of ICAM2-positive cells in resistant versus sensitive tumors (21.0% vs. 9.3%; Fig. 2C). This increase was confirmed at the protein level in both fulvestrant-resistant mouse model-derived cells and MCF7 FulR cells (Fig. S2B). We next stratified resistant BC cells (scRNA-seq data) into ICAM2-negative (log_2_ expression = 0) and ICAM2-positive (log_2_ expression > 0) groups. Subsequently, KEGG pathway analysis showed significant enrichment of OXPHOS in ICAM2-positive cells (Fig. S2C). Consistent with this, violin plots confirmed marked upregulation of OXPHOS and mitochondrial-related genes in the ICAM2-positive population (Fig. 2D).

To further investigate whether there is a direct link between ICAM2 overexpression and high OXPHOS, we sorted ICAM2-positive and ICAM2-negative cancer cells from resistant tumors for bulk RNA-seq. Differential expression analysis identified 191 upregulated and 1308 downregulated genes in ICAM2-positive cells (|log_2_FC | > 1, *p* < 0.05) (Fig. S2D). KEGG enrichment showed that the upregulated genes were significantly associated with oxidative phosphorylation (Fig. 2E).

We then validated key OXPHOS components by RT-qPCR and found that expression of mitochondrial respiratory chain subunits—including *MT-ND2* (complex I), *MT-CYB* (complex III), *MT-CO1/MT-CO2* (complex IV), and *ATP8* (ATP synthase)—was markedly elevated in ICAM2-positive resistant cells (Fig. 2F). Ultrastructurally, ICAM2-positive cells displayed elongated mitochondria with densely packed cristae (Fig. 2G). Functional Seahorse assays confirmed that these cells exhibited accelerated oxygen consumption rates, indicating heightened mitochondrial oxidative respiration (Fig. 2H). Taken together, these results demonstrate that ICAM2-positive cancer cells, which arise during acquired endocrine resistance, operate in a high-OXPHOS state that promotes resistance.

### ICAM2-positive cancer cells are essential for endocrine resistance

**Fig. 3:**
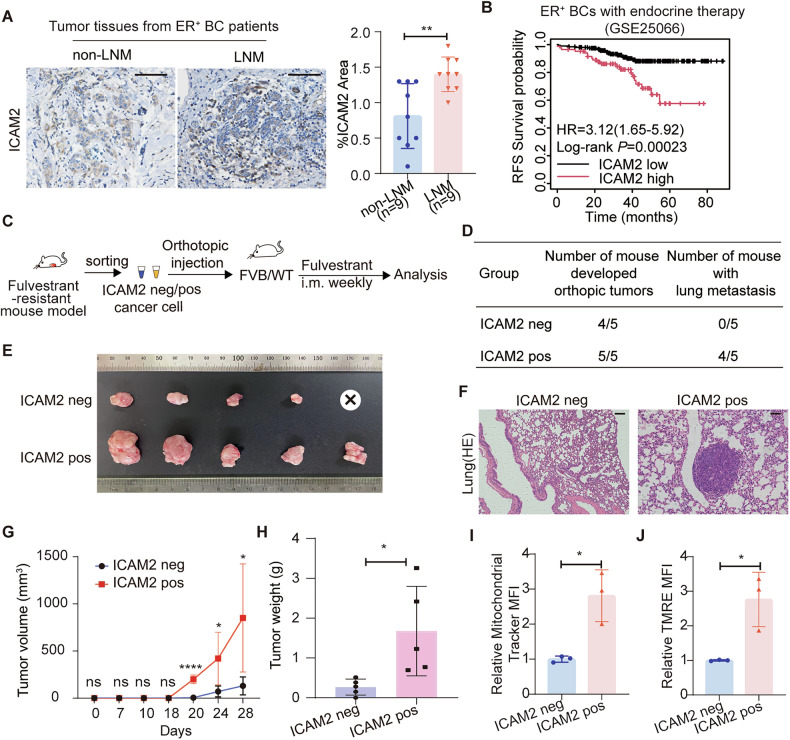
ICAM2-positive cancer cells play a pivotal role in acquired resistance to endocrine therapy. **A** Immunohistochemical analysis of ICAM2 expression in clinical ER^+^ BC samples. Representative images and quantification of ICAM2-positive areas in tumor tissues from patients with lymph node metastasis (LNM, *n* = 9) and without lymph node metastasis (non-LNM, *n* = 9). Scale bar, 20 µm. **B** Survival probability analysis of ICAM2 low (*n* = 199) and ICAM2 high (*n* = 84) ER^+^ BC patients treated with endocrine therapy (GSE25066). Hazard ratio (HR). The *p-*values were determined by the log-rank test. **C** Schematic diagram of the orthotopic xenograft procedure. ICAM2 negative (ICAM2 neg) and ICAM2 positive (ICAM2 pos) cancer cells, sorted from tumor tissues of fulvestrant resistant mouse model, were orthotopically implanted. Fulvestrant treatment weekly (50 mg/kg, intramuscular) commenced. **D** Incidence of orthotopic tumor formation and metastasis derived from (**C**). **E** Images of the developed orthotopic xenografts from (C). **F** Representative HE staining of lung tissue from (**C**). Scale bar, 50 µm. **G** Quantification of orthotopic xenograft tumor volume in (**E**). *n* = 5 per group. Statistical analysis was performed using Student’s *t*-test. **H** Quantification of orthotopic xenograft tumor weight in (**E**). (**I, J**) Flow cytometry analysis of cancer cells from (**E**). Cells stained with MitoTracker Green (**I**) or TMRE (**J**) were assayed, and relative median fluorescence intensities are shown (*n* = 5, each group). MFI: median fluorescence intensities. ns: no significance, *p < 0.05, **p < 0.01, ****p < 0.0001.

We further investigated the clinical relevance of ICAM2 expression in endocrine resistance. Immunohistochemical analysis revealed that primary ER^+^ BCs from patients with lymph node metastasis (LNM, *n* = 9) contained significantly larger ICAM2-positive areas than those without metastasis (non-LNM, *n* = 9; Fig. 3A), linking ICAM2 expression to metastatic progression. Clinically, in a cohort of ER^+^ BC patients (GSE25066), stratification by median ICAM2 expression revealed that high-ICAM2 cases (*n* = 84) had significantly reduced survival probabilities after endocrine therapy than low-ICAM2 cases (*n* = 199; Fig. 3B). These findings suggest ICAM2 as a biomarker predictive of poor therapeutic response in endocrine-treated ER^+^ BC.

To elucidate the functional contribution of ICAM2-positive cells in endocrine resistance, we orthotopically transplanted ICAM2-negative (ICAM2 neg) and ICAM2-positive (ICAM2 pos) cancer cells sorted from resistant tumors into wild-type FVB mice (Fig. 3C). Strikingly, under concomitant fulvestrant treatment, tumors derived from ICAM2 pos cancer cells exhibited significantly higher engraftment rates and a greater propensity for lung metastasis than those from ICAM2 neg cancer cells (Fig.3D–F). ICAM2 pos-derived xenografts also developed larger tumor volumes and heavier tumor weights than ICAM2 neg-derived tumors despite continuous fulvestrant administration (Fig.3G, H). Flow cytometry of cancer cells isolated from these xenografts revealed that primary cancer cells from ICAM2 pos tumors possessed significantly elevated mitochondrial mass (Fig. 3I) and increased mitochondrial membrane potential (Fig. 3J) compared to those from ICAM2 neg tumors. These results functionally link the ICAM2 positive state to enhanced mitochondrial activity and a therapy-resistant, metastatic phenotype in vivo.

### ICAM2 depletion impairs OXPHOS in endocrine-resistant cancer cells

**Fig. 4:**
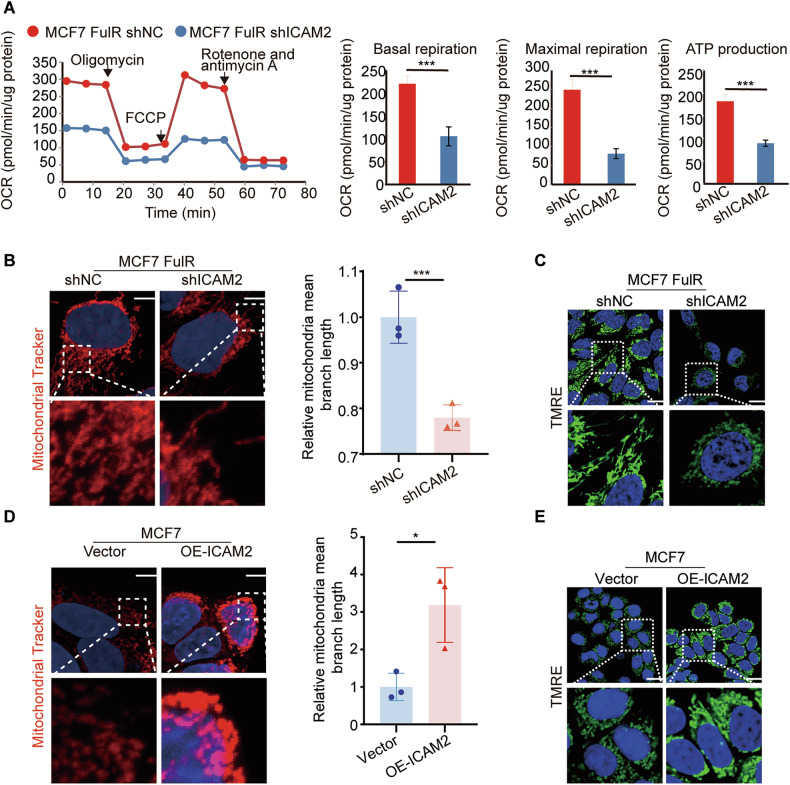
ICAM2 modulates mitochondrial function, thereby influencing OXPHOS. **A** The oxygen consumption rate (OCR) levels were measured in MCF7 FulR cells following transfection with shNC or shICAM2 lentivirus. Basal respiration, maximal respiration, and ATP production are shown individually. *n* = 5 for each group. All data are shown as mean ± SEM. **B** Representative fluorescence images of mitochondria (red) in MCF7 FulR cells transfected with shNC or shICAM2. Scale bar, 10 µm. **C** TMRE signals in MCF7 FulR shNC/shICAM2 cells were detected by fluorescence microscopy. Scale bar, 20 µm. **D** Representative fluorescence images of mitochondria (red) in MCF7 cells transfected with empty vector control or ICAM2-overexpressing plasmid (OE-ICAM2). Scale bar, 10 µm. **E** TMRE signals in MCF7 Vector/OE-ICAM2 cells. Scale bar, 20 µm. *p < 0.05, ***p < 0.001.

To directly assess whether ICAM2 is required to sustain the high-OXPHOS state in endocrine-resistant cells, we knocked down ICAM2 in MCF7 FulR cells using lentiviral shRNA (Fig. S3A). Mitochondria stress assays revealed that ICAM2 knockdown significantly impaired oxygen consumption rates (OCR), indicating a reduction in OXPHOS capacity (Fig. 4A). In line with this functional dependence, ICAM2 depletion fragmented the mitochondrial network and decreased mitochondrial membrane potential in MCF7 FulR cells (Figs. 4B, C, S3B, C), whereas its overexpression (OE-ICAM2) promoted mitochondrial clustering and membrane potential in MCF7 cells (Figs. S3D, 4D, E). Moreover, CCK-8 assays showed that ICAM2 knockdown restored sensitivity to fulvestrant (Fig. S3E).

For in vivo validation, we orthotopically implanted MCF7 FulR shNC or shICAM2 cells into female nude mice and administered weekly fulvestrant (Fig. S3F). Longitudinal imaging showed attenuated tumor growth in the shICAM2 group under treatment (Fig. S3G), and endpoint analysis confirmed a pronounced reduction in tumor incidence compared to controls (Fig. S3H). Together, these results establish that ICAM2 is essential for maintaining OXPHOS and thereby supporting acquired endocrine resistance in BC.

### The ICAM2-Dynein-complex I interaction orchestrates mitochondrial function

**Fig. 5:**
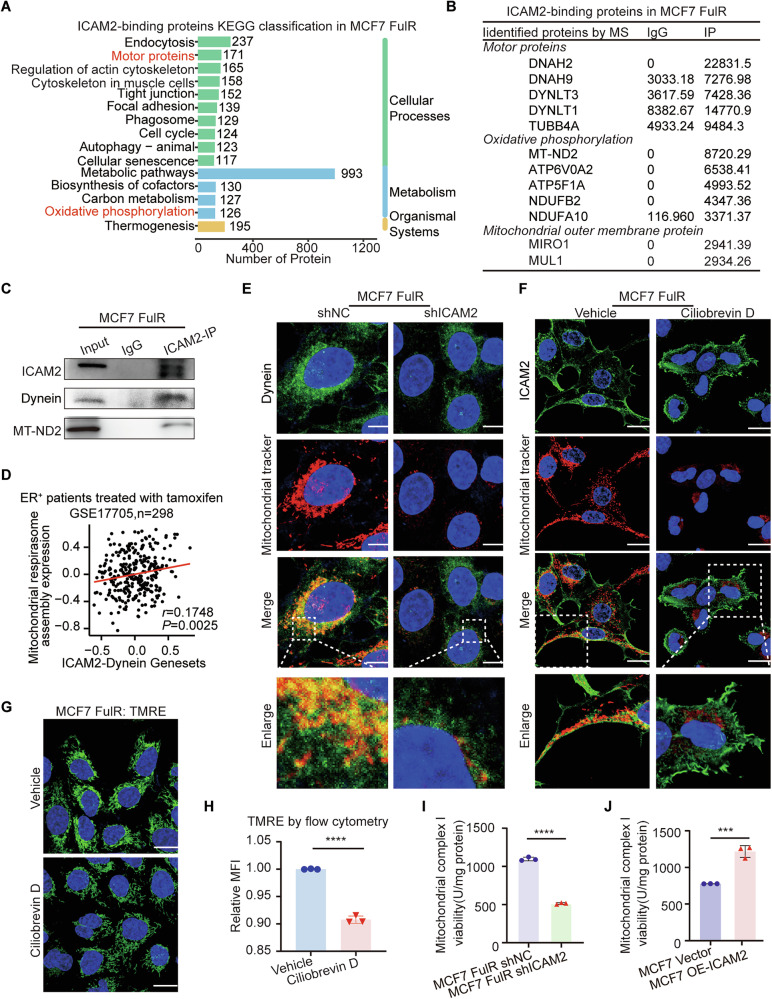
ICAM2-Dynein-dependent mitochondrial trafficking maintains OXPHOS. **A** KEGG analysis of ICAM2-interacting proteins in MCF7 FulR cells was identified by a co-immunoprecipitation-based mass spectrometry assay. Peptides were immunoprecipitated with the ICAM2 antibody. **B** Proteins co-immunoprecipitating with ICAM2 and associated with motor proteins, oxidative phosphorylation, or mitochondrial outer membrane protein identified by LC-MS/MS in MCF7 FulR cells. **C** The interaction between ICAM2, DYNLT3, and MT-ND2 in MCF7 FulR cells was detected by Co-IP. Cell lysates were immunoprecipitated using anti-ICAM2 antibodies and subjected to a Western blot with specified antibodies. **D** Positive correlation between mitochondrial respirasome assembly and co-expression of ICAM2 and Dynein complex in ER^+^ BC patients receiving tamoxifen therapy (GSE17705; *n* = 298). **E** Representative immunofluorescence (IF) images depicting co-localization of Dynein (green) and mitochondria (red) in MCF7 FulR cells transfected with either non-targeting control shRNA (shNC) or ICAM2-targeting shRNA (shICAM2). Scale bar, 10 µm. **F** Representative IF images depicting co-localization of ICAM2 (green) and mitochondria (red) in MCF7 FulR cells treated with ciliobrevin D (5 μM) for 24 hours. Scale bar, 20 µm. **G** TMRE signals in MCF7 FulR cells treated with ciliobrevin D (5 μM) for 24 hours were detected by fluorescence microscopy. Scale bars, 20 µm. **H** Flow cytometry analysis of relative TMRE MFI in MCF7 FulR cells treated with ciliobrevin D (5 μM) for 24 hours (*n* = 3, each group). **I** The mitochondrial complex I activities were measured in MCF7 FulR shNC/shICAM2 cells. Data: mean ± SD (*n* = 3). **J** The mitochondrial complex I activities were measured in MCF7 vector/OE-ICAM2 cells. Data: mean ± SD (*n* = 3). ***p < 0.001, ****p < 0.0001.

To investigate the mechanism linking ICAM2 to elevated OXPHOS, we performed co-immunoprecipitation followed by mass spectrometry (Co-IP/MS) in MCF7 FulR cells to identify ICAM2-interacting proteins. KEGG pathway analysis of these interactors showed significant enrichment for motor protein complexes and OXPHOS components (Fig. 5A, S4A). Notably, subunits of the motor protein Dynein (DNAH2, DNAH9, DYNLT3) and several mitochondrial components —including the complex I subunit MT-ND2 and the mitochondrial outer membrane protein MIRO1—were among the top hits (Fig. 5B). The direct physical interaction between endogenous ICAM2, Dynien, and MT-ND2 in MCF7 FulR cells was corroborated by Co-IP/Western blot (Fig. 5C), and immunofluorescence further confirmed ICAM2 and MT-ND2 co-localization in ICAM2-positive-derived xenografts (Fig. S4B). Supporting the molecular interaction, transcriptomic profiling of clinical metastatic BC (GSE209998; *n* = 79) revealed a strong positive correlation between *ICAM2* and *Dynein* subunit gene expression (Fig. S4C). In a clinical cohort of tamoxifen-treated ER^+^ BCs (GSE17705, *n* = 298), co-expression of ICAM2 and Dynein complex genesets was positively correlated with enhanced mitochondrial respirasome assembly (Figs. 5D, S4D). Given the established role of the Dynein complex in mitochondrial trafficking and positioning [25], these findings prompted the hypothesis that the ICAM2–Dynein interaction supports mitochondrial function in endocrine-resistant cells. Consistent with this, ICAM2 knockdown in MCF7 FulR cells diminished Dynein expression and abrogated Dynein-mitochondria co-accumulation (Fig. 5E). To test causality, pharmacological inhibition of Dynein with ciliobrevin D was employed. Results showed that Dynein inhibition reduced both ICAM2-mitochondrial co-localization and mitochondrial membrane potential, while ICAM2 expression remained unchanged (Fig. 5F–H). Collectively, these findings delineate a mechanism wherein ICAM2-Dynein interaction is essential for maintaining mitochondrial activity in endocrine-resistant BC cells.

To identify the mitochondrial effectors linking ICAM2–Dynein to OXPHOS, we intersected ICAM2-interacting proteins (Co-IP/MS) with genes upregulated in ICAM2-positive resistant cancer cells. A mitochondrial complex I subunit MT-ND2 emerged as the sole overlapping candidate (Fig. S4E), implicating complex I - rather than complexes III, IV, or ATP synthase- as the key functional partner of ICAM2, despite the upregulated expression of other subunits observed in ICAM2-positive resistant cancer cells (Fig. 2F). Therefore, we focused subsequent functional studies on complex I in endocrine-resistant BC cells. Functionally, ICAM2 knockdown in MCF7 FulR cells reduced mitochondrial complex I activity (Fig. 5I), whereas overexpression of ICAM2 in parental MCF7 cells significantly enhanced it (Fig. 5J). These results confirmed mitochondrial complex I as a critical functional link between ICAM2 and OXPHOS in endocrine resistance. Collectively, our results demonstrate that the ICAM2–Dynein-complex Ⅰ axis is essential for sustaining OXPHOS in endocrine‑resistant BC cells.

### Inhibition of mitochondrial complex I restores endocrine therapy sensitivity

**Fig. 6:**
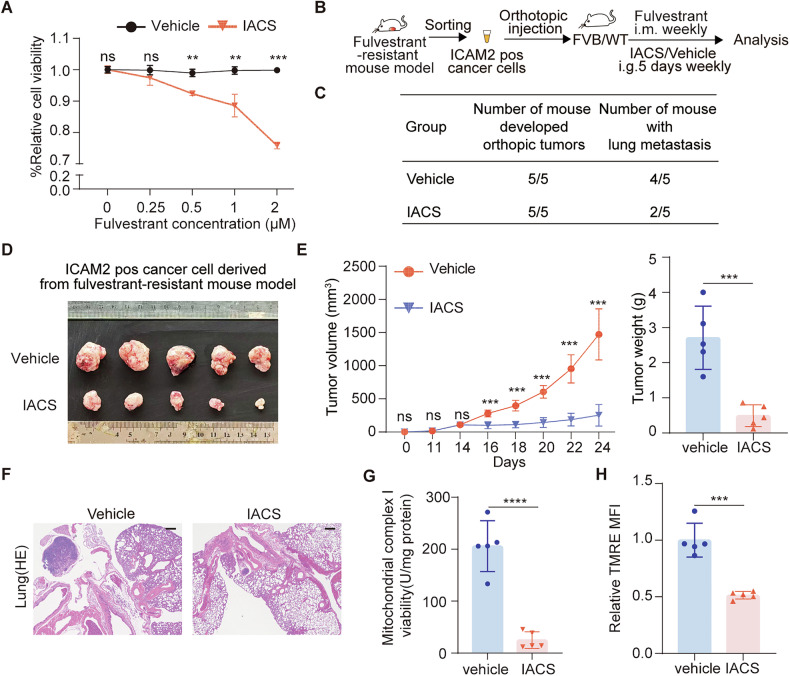
Inhibition of mitochondrial complex I overcomes fulvestrant resistance. **A** Cell viability after 72 hours co-treatment with inhibitor of complex I, IACS-10759 (IACS, 10^−^^10^M) or vehicle plus fulvestrant (dose range). **B** In vivo experimental design diagram. ICAM2-positive cells from a fulvestrant-resistant mouse model were orthotopically implanted into a female FVB wild-type mouse. At tumor volumes of ~100 mm³, mice were randomized to vehicle (i.g., 5 days/week) and IACS (2.5 mg/kg, i.g., 5 days/week) groups. Both groups received concurrent fulvestrant (50 mg/kg, i.m., once weekly). **C** Orthotopic tumor and metastasis incidence from the indicated experimental groups (**B**). **D** Images of the developed orthotopic xenografts from (**B**). **E** Quantification of orthotopic xenograft tumor volume and weight in (**B**). **F** Representative HE staining of lung tissue from (**B**). Scale bar, 50 μm. **G** The mitochondrial complex I activities were measured in tumor tissues from (**B**). **H** FACS analyzed relative TMRE MFI reflecting mitochondrial membrane potential measured in tumor tissues from the indicated experimental groups (**B**). Values are mean ± SD (*n* = 5 tumors each group). ns: no significance, ***p < 0.001, ****p < 0.0001.

To determine whether complex I directly contributes to endocrine resistance, we first investigated the therapeutic effect of combining the complex I inhibitor IACS-10759 with fulvestrant in resistant BC cells. This combination significantly enhanced sensitivity compared with fulvestrant alone (Fig. 6A). We then asked whether inhibiting complex I could reverse therapy resistance in vivo. Primary ICAM2 pos cancer cells isolated from resistant tumors were orthotopically implanted into wild-type FVB mice (Fig. 6B). Compared with the vehicle plus fulvestrant group, the IACS/fulvestrant combination markedly suppressed primary tumor growth, as evidenced by reduced tumor volume and weight (Fig. 6C–E), together with a lower incidence of lung metastasis (Fig. 6F). Mechanistically, tumors from the combination group exhibited decreased mitochondrial complex I activity (Fig. 6G) and reduced mitochondrial membrane potential (TMRE fluorescence; Fig. 6H). Together, these results demonstrate that pharmacologic inhibition of complex I restores sensitivity to endocrine therapy in resistant BC.

### ER repression drives ICAM2 upregulation in endocrine resistance

**Fig. 7:**
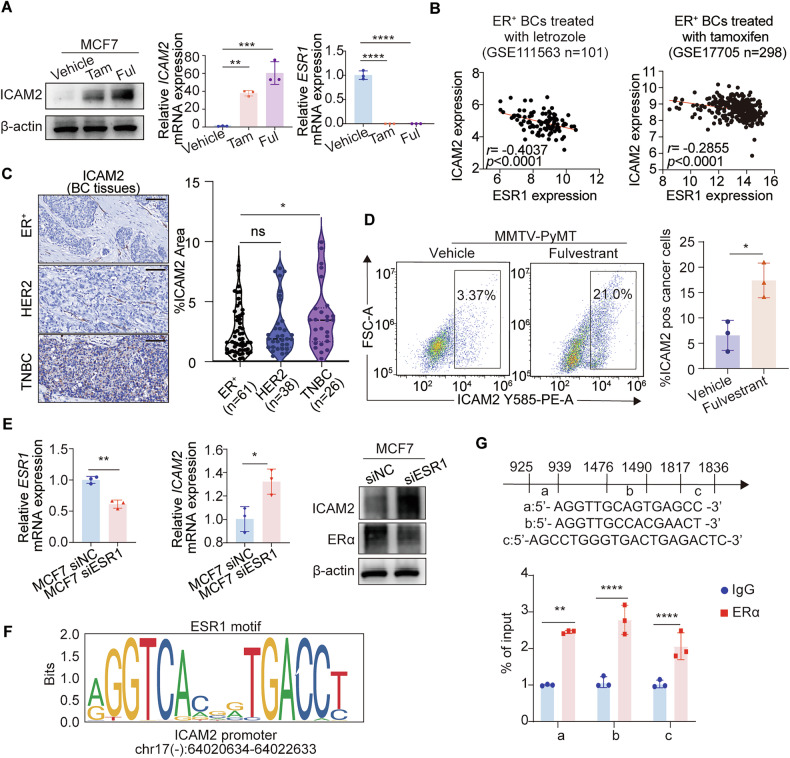
ERα inhibition upregulates ICAM2 in fulvestrant-resistant cells. **A** Western blot analysis of ICAM2 and RT-PCR analysis of ICAM2 and ESR1 expression levels in MCF7 cells treated with tamoxifen and fulvestrant for 72 hours. **B** Negative correlation between ICAM2 and ESR1 expression in ER^+^ breast cancer patients treated with letrozole (GSE111563) or tamoxifen (GSE17705). **C** Quantification of ICAM2 expression (measured by H-score) in a tissue microarray containing luminal (ER^+^, *n* = 61), HER2-positive (HER2, *n* = 38), and triple-negative (TNBC; *n* = 26) human BC tissues. Data represent mean ± SEM. **D** Flow cytometry analysis of ICAM2-positive (pos) cancer cells from MMTV-PyMT mice treated with fulvestrant (fulvestrant, 50 mg/kg, i.m., once weekly for 8 weeks) or vehicle control (vehicle, *n* = 3 mice/group). Data represent the mean percentage ± SEM of the CD45^-^ EpCAM^+^ICAM2^+^ cell population. **E** RT-PCR and Western blot analysis of ICAM2 and ESR1 expression levels in MCF7 cells transfected with siNC (negative control siRNA) or siESR1 (ESR1-targeting siRNA). Levels were normalized to β-actin. **F** Predicted ERα binding sites in the ICAM2 promoter. Sequence predictions generated using the JASPAR database. **G** ChIP-qPCR analysis assessing ERα binding to three regions (−925 to −939, −1476 to −1490, −1817 to −1836 bp) in the ICAM2 promoter in MCF7 cells. Data represent mean ± SEM (*n* = 3). *p < 0.05, **p < 0.01, ***p < 0.001, ****p < 0.0001.

Given the sustained increase in ICAM2 observed in resistant BC cells following long-term ER inhibition, we hypothesized that ER repression drives its expression during the acquisition of endocrine resistance. To test this, we first analyzed the correlation between ICAM2 and ERα. Interestingly, ER inhibition triggers a marked upregulation of ICAM2 in BC cells upon short-term antiestrogen treatment (fulvestrant or tamoxifen) (Fig. 7A). This suggests a potential inverse regulatory relationship between ICAM2 and ER activity. This negative correlation is consistently observed across multiple clinical settings: ICAM2 mRNA levels inversely correlate with ESR1 expression in the TCGA BC cohort (*n* = 1100) (Fig. S5A); its expression is highest in ER-negative TNBC and lowest in ER-positive luminal-like subtypes (Fig. S5B), further associating it with ER pathway inactivation. Furthermore, in ER^+^ BC patients treated with letrozole or tamoxifen, ICAM2 remains inversely correlated with ESR1 (Fig. 7B). Notably, immunohistochemical analysis confirms the inverse correlation at the protein level, the expression level of ICAM2 is highest in ER-negative TNBC and lowest in ER-positive subtypes (Fig. 7C, Table S2).

To validate this inverse regulation mechanistically, we employed three complementary approaches. First, the ERα-low/ICAM2-high phenotype was observed in fulvestrant-resistant MCF7 FulR cells versus parental (Fig. S5C, D). This relationship held in vivo, where fulvestrant treatment in MMTV-PyMT mice significantly increased the proportion of ICAM2-positive tumor cells (Fig. 7D). Finally, direct siRNA-mediated ESR1 knockdown in MCF7 cells upregulated ICAM2 (Fig. 7E), suggesting ERα suppression as a direct driver of ICAM2 expression.

To test whether ERα directly represses ICAM2 transcription, we first used JASPAR to predict potential ERα binding sites on the ICAM2 promoter, identifying three candidate regions (925–939, 1476–1490, and 1817–1836; Fig. 7F). Subsequent ChIP-qPCR analysis in MCF7 FulR cells with an ERα antibody confirmed its specific binding to all three sites (Fig. 7G), thereby establishing ERα as a direct transcriptional repressor of ICAM2 in endocrine-responsive contexts.

### High ICAM2 expression correlates with poorer treatment outcomes in ER-positive BC patients

**Fig. 8:**
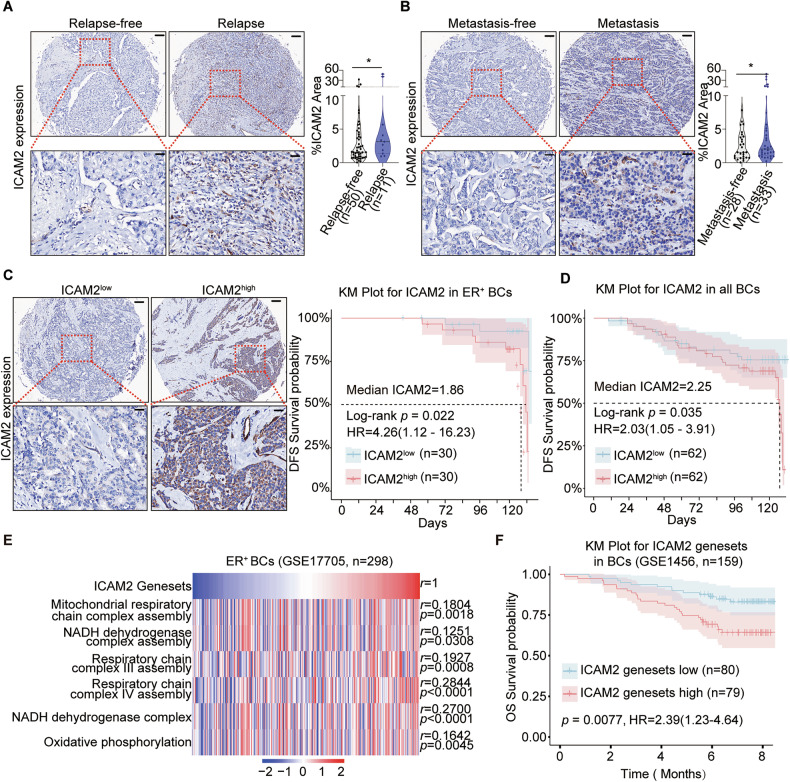
ICAM2^high^ signature is associated with unfavorable treatment outcomes in ER^+^ breast cancer patients. **A** Representative IHC images (left) and quantification of ICAM2-positive frequency (right) in primary tumors from patients who later relapsed (relapse, *n* = 11) versus those who did not (relapse-free, *n* = 50). Scale bar, top:100 μm, bottom:20 μm. The dotted lines show the median and quartile values. **B** Representative IHC images (left) and quantification of ICAM2-positive cell frequency (right) in primary tumors from patients who developed metastasis (metastasis, *n* = 33) versus those without metastasis (metastasis-free, *n* = 28). Scale bar, top:100 μm, bottom:20 μm. The dotted lines show the median and quartile values. **C** Representative ICAM2 IHC images (left) and disease-free survival (DFS) analysis (right) in ER^+^ BCs (ICAM2^low^, *n* = 30; ICAM2^high^, *n* = 30). Scale bar, top:100 μm, bottom:20 μm. **D** DFS analysis in all BC patients (ICAM2^low^, *n* = 62; ICAM2^high^, *n* = 62). **E** Heatmap depicting correlation signature patterns of the ICAM2 gene sets in tamoxifen-treated human ER^+^ breast cancer (GSE17705; *n* = 298). **F** Overall survival (OS) analysis in breast cancer patients stratified by median ICAM2 genesets (GSE1456, ICAM2 genesets high, *n* = 79; ICAM2 genesets low, *n* = 80). ns (no significance), *p < 0.05.

To investigate the clinical significance of ICAM2 expression in BC progression, we conducted immunohistochemical analysis of ICAM2 levels using a tissue microarray containing 125 human BC specimens representing diverse molecular subtypes. These primary tumor samples were collected from patients with or without metastatic disease at initial diagnosis, as well as from those with or without recurrence within 10 years after surgery (Table S2). In ER^+^ BC cases, immunohistochemistry revealed higher ICAM2 expression in tumors from patients who experienced relapse (*n* = 11) or metastasis (*n* = 33), compared to those from relapse-free patients (*n* = 50) or metastasis-free(n=28)(Fig. 8A, B). To further assess the prognostic relevance, ER^+^ samples were stratified into “high” and “low” ICAM2 groups based on median staining intensity. Results showed that patients with elevated ICAM2 expression exhibited a significantly shorter disease-free survival (DFS) in both ER⁺ (Fig. 8C, median ICAM2 intensity: 1.86) and overall BC populations (Fig. 8D, median ICAM2 intensity: 2.25), suggesting a potential role of ICAM2 in promoting disease recurrence and progression in ER^+^ BC.

Additionally, to define a mechanistic gene signature associated with ICAM2, we integrated 20 genes that were consistently upregulated in both resistant tumor (proteomic data) and ICAM2-positive cancer cells (bulk RNA-seq). This integrated set was compiled into an ICAM2-related signature gene set for evaluating its association with adverse tumor outcomes. Analysis of clinical ER^+^ BC data (GSE17705) showed that expression of this signature positively correlated with activity of the mitochondrial respiratory chain complex and with oxidative phosphorylation pathways (Fig. 8E). To further assess its clinical relevance, samples were stratified into “ICAM2 genesets high” and “ICAM2 genesets low” groups based on median signature expression. Kaplan–Meier analysis revealed that patients with higher signature expression had significantly shorter disease-free survival (Fig. 8F). Together, these results suggest that the ICAM2^high^ signature is linked to enhanced oxidative phosphorylation and may thereby contribute to tumor progression and poorer clinical outcomes.

## Discussion

The mechanisms underlying endocrine resistance remain incompletely understood. Among established treatment resistance pathways, OXPHOS-driven mitochondrial dysregulation represents a key adaptive strategy in cancer [26]. While targeting OXPHOS shows preclinical potential—by alleviating hypoxia and enhancing immunogenic cell death—its clinical translation remains uncertain and may be context-dependent. A critical therapeutic paradox lies in the risk that OXPHOS inhibition fails to reverse, and may even exacerbate, glycolytic acidosis in the tumor microenvironment. The central unresolved question is how adaptive signaling dynamically upregulates OXPHOS to fuel tumor survival and confer resistance?

Utilizing an endocrine-resistant tumor model, our integrated multi-omics analysis revealed that endocrine-resistant ER^+^ BC cells exhibit mitochondrial gene upregulation and a metabolic shift towards OXPHOS dependency. This metabolic phenotype, validated by ultrastructural, functional, and biochemical assays, aligns with emerging evidence that OXPHOS upregulation serves as a critical adaptive mechanism in endocrine-resistant BCs. Consistent with our findings, OXPHOS has been implicated in endocrine resistance by recent studies [17–19]. Despite the association between OXPHOS and endocrine resistance, how cancer cells adapt to therapy by boosting OXPHOS and reprogramming mitochondrial function for survival remains to be clarified.

For the first time, in this study, we identify the adhesion molecule ICAM2—previously linked to immune trafficking and metastasis [22, 27]—as a key membrane marker associated with OXPHOS activation in endocrine-resistant BC. Despite its known roles, ICAM2 specifically marks OXPHOS-high resistant tumors. ICAM2^+^ resistant cells exhibit hyper-functional mitochondria, characterized by dense cristae and elevated oxygen consumption, whereas ICAM2 knockdown diminishes mitochondrial content and complex I activity. Functionally, ICAM2 drives in vivo resistance and metastasis, maintaining high mitochondrial mass/potential—effects reversed by its knockdown. Clinically, high ICAM2 correlates with poorer outcomes in ER^+^ patients, highlighting its dual role as a functional mediator and potential biomarker of OXPHOS-dependent resistance.

We further elucidated a novel mechanism underlying endocrine therapy resistance: an ICAM2-DYNLT3 axis promoting OXPHOS activation in ER^+^ BC. Co-IP-based MSI analyses demonstrate that ICAM2 physically interacts with the motor protein Dynein complex component DYNLT3 and subunits of the electron transport chain complex I MT-ND2. Disruption of this interaction, either through pharmacological inhibition of Dynein or ICAM2 knockdown, resulted in diminished mitochondrial presence at the cellular periphery in resistant cells. Although previous studies reported that the intracellular domain of ICAM2 often links to the cytoskeleton through interaction with α-actinin [28] and with members of the ERM family [29], controlling endothelial junctions [23], its interaction with motor proteins has not been revealed before. It is reported that, in response to mitochondrial dysfunction, actin polymerization exhibits a dynamic and context-dependent regulatory role, which enables both structural adaptation and precise, often opposing motility effects—via its own filaments or motors like myosin 19 [30], though their mechanisms are largely unknown. Our study links the membrane molecule ICAM2 to motor proteins and complex I in BCs with acquired endocrine resistance. This reveals a coordinated pathway that fine-tunes the OXPHOS-high metabolic phenotype, thereby driving adaptive treatment resistance.

Critically, the reliance of ICAM2^+^ cells on OXPHOS, particularly complex I activity, presents a therapeutic vulnerability. The synergistic efficacy observed with the complex I inhibitor IACS-10759 combined with fulvestrant, significantly suppressing tumor growth and metastasis in preclinical models, validates targeting this metabolic axis as a promising strategy to overcome ICAM2-driven resistance. Although OXPHOS inhibition via complex I inhibitors (e.g., IACS-010759, metformin) is a promising preclinical strategy in cancers like renal cell carcinoma [31], its clinical application is challenged by serious on-target toxicities and a narrow therapeutic window [32]. In contrast, our work reveals that endocrine-resistant BC depends on a specific ICAM2-Dynein-OXPHOS axis. Here, ICAM2 depletion selectively disrupts OXPHOS, exploiting a metabolic dependency tied to tumor progression and poor prognosis, thereby presenting a potentially more targeted and tolerable therapeutic approach.

Notably, we identified the mechanism driving ICAM2 overexpression in resistant BC: ERα loss de-represses ICAM2 transcription. This is supported by an inverse clinical correlation between ER and ICAM2, experimental upregulation of ICAM2 upon ERα knockdown, and direct evidence of ERα binding to the ICAM2 promoter via ChIP-PCR. Thus, ERα downregulation—a hallmark of endocrine resistance—relieves this repression, ultimately driving ICAM2 overexpression.

Collectively, this work revealed ICAM2 as both a critical metabolic regulator and a potential biomarker signifying emerging treatment resistance in ER^+^ BC. By uncovering the mechanism whereby ICAM2 recruits the Dynein motor complex via DYNLT3 to orchestrate mitochondrial positioning and bioenergetics, and by demonstrating how ERα loss derepresses ICAM2 expression, we have identified multiple strategies for therapeutic intervention. Future studies should prioritize the clinical validation of ICAM2 as a predictive biomarker and rigorously evaluate combinatorial strategies targeting OXPHOS to overcome resistance in defined patient subsets.

## Materials and Methods

### Antibodies and reagents

Antibodies and reagents utilized in this study are detailed in the key resource table (Table S3).

### Cell culture

The breast cancer cell line MCF7 was obtained from ATCC. Our laboratory construction generated the MCF7 FulR and MCF7 TamR cell lines [33]. All cell lines were routinely cultured at 37°C in a humidified incubator maintained at saturated humidity with an atmosphere of 5% CO₂. Complete growth media for all cell lines were supplemented with a penicillin/streptomycin solution at a final concentration of 1%. MCF7 cells were grown in MEMα medium (Gibco, #12571) supplemented with 10% FBS (Gibco, #10270), 1× NEAA (Gibco, #11140), and 0.01 mg/mL insulin (Sigma, #I0516). Authentication of cell lines was performed using short tandem repeat genotyping, and the absence of Mycoplasma contamination was confirmed using the Universal Mycoplasma Detection Kit (ATCC).

### Mice

For animal studies, MMTV-PyMT (Fvb/NJgpt) transgenic mice, FVB/NJgpt/WT, and BALB/c nude mice were sourced from GemPharmatech Co., Ltd. (Nanjing, China), with all protocols reviewed and approved by the Institutional Animal Care and Use Committee of Shanghai Sixth People’s Hospital (No.2022-0356; No:2024-0394). The fulvestrant-resistant mouse models were established as previously reported [24]. Tumors were digested into single-cell suspensions in HBSS/2% FBS, incubated with antibodies, and analyzed by flow cytometry. Subsequently, EpCAM^+^/CD45^-^/ICAM2ⁿᵉᵍ/ᵖᵒˢ cancer cell populations sorted by FACS were randomly orthotopically injected in cold 50% Matrigel (BD Biosciences, USA) into mammary fat pads of 3–4-week-old female FVB/WT mice (*n* = 5/group). When tumors reached ~100 mm³ (volume = width^2 × length × 0.52), mice randomly received weekly intramuscular fulvestrant (50 mg/kg) alongside IACS-010759 (IACS; 2.5 mg/kg/day) or vehicle administered by oral gavage for five consecutive days weekly (*n* = 5/group). IACS was dissolved in DMSO (15 mg/mL) and diluted in 0.5% methylcellulose suspension (100 μL dose). Mice were euthanized at tumor volume ≥2000 mm^3^, with tumor and lung samples processed for histology HE staining following fixation and paraffin embedding. In a parallel study, female athymic BALB/c nude mice (4–6 weeks) underwent randomly orthotopic mammary fat pad injections of MCF7 FulR shNC or MCF7 FulR shICAM2 cells (1 × 10^6^ cells) mixed with Matrigel (*n* = 5/group).

### Integrated proteomics and metabolomics analysis

Cancer cells isolated by FACS (BD FACSAria III, >99% purity, 1×10⁶ cells/sample) were immediately processed for multi-omics analysis by MetWare Biotechnology Co., Ltd (Wuhan, China). For proteomics, sorted cells were lysed in lysis buffer (containing 8 M urea, 1 mM PMSF, and 2 mM EDTA), digested with trypsin (Promega), separated by nano-flow high-performance liquid chromatography, and subjected to DIA (Data-Independent Acquisition) mass spectrometry analysis using the Orbitrap Astral high-resolution mass spectrometer (Thermo Scientific). For metabolomics, cells were quenched in -40°C methanol/water (7:3 v/v) with isotopically labeled internal standards, followed by LC-QTOF-MS/MS. Raw proteomic data were processed via MaxQuant (the uniprotkb_proteome_UP000000589_mouse_54910_20240528.fasta database, FDR < 1%), while metabolomic features were extracted using XCMS and annotated against MetWare’ s in-house database MWDB ((including secondary spectra and retention time RT), the integrated DB-all public database (including Metlin, HMDB, KEGG, etc.), AI prediction libraries, and MetDNA.

### Transmission Electron Microscopy (TEM)

Cells were fixed in 2.5% glutaraldehyde solution, followed by removal of the fixative and three 15-minute rinses in 0.1 M phosphate buffer (pH 7.0). Subsequently, samples were post-fixed in 1% osmium tetroxide for 1–2 hours. The osmium tetroxide solution was carefully removed, and samples were rinsed three times (15 min each) in 0.1 M phosphate buffer (pH 7.0). Dehydration was performed using a graded ethanol series (30%, 50%, 70%, 80%, 90%, and 95%; 15 minutes per concentration), followed by two 20-minute changes in 100% ethanol. Samples were then transitioned to pure acetone for 20 minutes. Infiltration was initiated with a 1:1 (v/v) mixture of embedding resin and acetone for 1 hour, followed by a 3:1 (v/v) resin/acetone mixture for 3 hours. Samples were immersed in pure embedding resin overnight. Following infiltration, samples were embedded in fresh resin and polymerized at 70°C overnight. Ultrathin sections (70–90 nm) were cut using an ultramicrotome, stained sequentially with uranyl acetate (50% ethanol-saturated solution) and lead citrate, each for 5–10 minutes, air-dried, and examined using a transmission electron microscope. Imaging was performed on a JEOL JEM-1400Flash TEM operating at 120 kV equipped with a Gatan OneView CMOS camera. Images were acquired using Gatan Microscopy Suite software at magnifications ranging from 3000x to 30,000x. Representative images shown are from three independent biological replicates; for each replicate, multiple grids and sections were examined, and at least 10 representative cell profiles per condition were imaged.

### Flow cytometry

Live cells were incubated with 100 nM MitoTracker Green (Thermo Fisher, M7514), MitoTracker® Red CMXRos (Thermo Fisher, M7512), 100 nmol/L TMRE (Rhodamine 123, Invitrogen, R302), in detection buffer for 30 min at 37°C protected from light. Cells were washed twice with PBS (centrifugation: 300 × *g*, 5 min, 4°C) and analyzed immediately. At least 10,000 live single cells (gated by FSC-A/SSC-A, FSC-H/FSC-A, and DAPI exclusion) were acquired per sample. Flow Jo v10.8.2 (BD Biosciences) was used to calculate median fluorescence intensity (MFI). Positive populations for functional dyes were defined based on unstained controls. Antibody staining thresholds were set using isotype control antibodies (Mouse IgG1κ, BioLegend, #400120).

### Measurements of oxygen consumption rate (OCR)

OCR analysis was measured by Waldbronn, Germany Seahorse XFe96 analyzer from Seahorse Bioscience (Agilent, Delaware, USA) using the Seahorse XF Cell Mito Stress Test Kit (Agilent, Delaware, USA, 103015-100). Cells (1 × 10^4^ cells/well) were seeded in microplates (Agilent, Delaware, USA, 103729-100) and equilibrated in a CO2-free, buffered medium prior to real-time measurement of extracellular oxygen tension via solid-state fluorescent probes. The assay employs sequential injections of mitochondrial modulators - 1.5 μM oligomycin, 1.0 μM FCCP, and 0.5 μM rotenone/antimycin A - to interrogate specific respiratory parameters: basal respiration, ATP-linked respiration, and maximal respiratory capacity. Results are analyzed using Wave software, with respiratory parameters expressed as pmol/min/ μg protein, enabling quantitative comparison of mitochondrial dysfunction across experimental conditions.

### Human tumor tissues and tissue microarray analysis

Human tumor tissue analysis utilized a breast cancer tissue microarray (HBreD126Su10, Shanghai Superchip Biotech) comprising 125 breast lesions to evaluate ICAM2 expression frequency across molecular subtypes, with corresponding clinicopathological data obtained from medical records (Table S2). One sample was excluded due to missing DFS time, leaving 124 for survival analysis. Additionally, ICAM2 expression was assessed in a separate cohort of 18 human luminal-like breast tumor samples (non-lymph node metastasis, *n* = 9; lymph node metastasis, *n* = 9) collected from Shanghai Sixth People’s Hospital patients following ethical committee approval and informed consent.

### Immunohistochemistry (IHC)

Tissues were extracted, formalin-fixed, and paraffin-embedded. Paraffin sections for immunostaining underwent acetone fixation, air-drying, dewaxing, and dehydration. Antigen retrieval was conducted in Tris-EDTA buffer (pH 9.0) at 95°C for 20 min using steam heating. After PBS replenishment, sections were blocked with 5% BSA in PBS for 1 hour and incubated overnight at 4 °C with primary antibodies (Table S3). Detection employed goat anti-rabbit IgG-HRP (Invitrogen #G21234, 1:500) amplified with VECTASTAIN Elite ABC-HRP Kit (Vector Labs PK-6100). 3,3’-Diaminobenzidine (DAB) substrate (Dako K3468) provided brown staining monitored microscopically for 30–90 seconds, with development terminated by distilled water immersion. Staining intensity was quantified using ImageJ IHC Toolbox software.

### siRNA and Lentiviral transfection

Control and human ESR1-targeting siRNAs were designed by OBiO Technology (Shanghai, China). Cells were pre-incubated in antibiotic-free medium for 24 hours prior to transfection with 100 nM siRNA using the RiboFECT™ siRNA Transfection Kit (RiboBio Guangzhou, China) according to the manufacturer’s protocol, with knockdown efficiency subsequently evaluated by Western blot or qRT-PCR (siRNA sequences detailed in Table S3). Lentiviral particles encoding shRNA targeting human ICAM2 (hU6-MCS-CBh-gcGFP-IRES-puromycin) and ICAM2 overexpression (pcSLenti-CMV-ICAM2-3xFLAG-PGK-Puro-WPRE3) with both control particles were obtained from Genechem Technology. These viruses were transduced into MCF7 FulR cells using polybrene (5 μg/ml). Following a 24-hour incubation, stable cell populations were established through puromycin selection for 48 hours.

### CCK-8 assay

Cell proliferation was assessed using the CCK-8 assay (NCM Biotech, Shanghai, China, #C6005); 1 × 10³ cells/well in 96-well plate were treated with varying fulvestrant concentrations (0.25 μM–2 μM；48 h) for specified durations, then incubated with 10 μl CCK-8 reagent(10% v/v) at 37 °C for 2 h, and optical density at 450 nm was measured using a BioTek Epoch 2 microplate reader (Agilent, USA).

### Immunofluorescence

For immunofluorescence analysis, cells were fixed with 4% paraformaldehyde (Sigma-Aldrich P6148), permeabilized using 0.25% Triton X-100 (Sigma-Aldrich T8787), and blocked with 5% bovine serum albumin (BSA; Sigma-Aldrich A7906). Subsequently, cells were incubated overnight at 4 °C with primary antibodies (Table S3) diluted in PBS containing 1% BSA, followed by incubation with secondary antibodies (Table S3) for 1 hour at room temperature. Nuclei were counterstained with DAPI (Thermo Fisher D1306). Fluorescence images were acquired using a Nikon A1R confocal microscope (Nikon Instruments, Tokyo, Japan).

### RNA sequencing and microarray data analysis

RNA sequencing was performed on RNA extracted via the phenol-chloroform method from FACS-sorted EpCAM^+^/CD45^-^/ICAM2^neg/pos^ cancer cells derived from fulvestrant-resistant tumors. Sequencing libraries were prepared using the Illumina TruSeq RNA kit and sequenced on an Illumina HiSeq XTen system. Significant biological enrichments were identified through Gene Ontology and KEGG pathway analyses employing a hypergeometric test with FDR correction (*p* < 0.05). Gene expression profiles of paired human breast tumors were downloaded from GEO (Table S3); Log2-transformed data were collapsed to one probe per gene using GenePattern and subsequently converted to z-scores. Analysis of human tumor datasets was conducted as previously described [34], utilizing the GSVA algorithm to assign enrichment scores for each sample within defined gene sets. This included the ICAM2 geneset (*ICAM2, ATP8A1, ATP8A2, ATP8B1, ATP6V0B, ATP6V0C, ATP6V1A, MAPT, SPRY4, BDH1, PRPS1, DYNLT3, PLVAP, ALDOC, ESR1, PYCR1, MPC2, SPHK1, DCXR, ADH1*), which was derived from genes upregulated in IP-ICAM2 versus IP-IgG cells combined with proteomics data.

### Western blot

Total cell lysates were prepared, and equivalent amounts of protein were separated by SDS-PAGE and transferred to PVDF membranes (Millipore IPVH00010). Membranes were then blocked with 5% non-fat dry milk in TBST for 1 hour, followed by incubation with primary antibodies (Table S3) overnight at 4 °C. After washing with TBST (containing Tween-20; Sigma-Aldrich), membranes were incubated with HRP-conjugated secondary antibodies (Table S3) for 1 hour at room temperature. Protein bands were visualized using ECL Plus reagent (Thermo Fisher 32132), and signals were captured with an Amersham Imager 680 (Cytiva). All Western blot experiments were performed in triplicate. Uncropped immunoblot gels are shown in Supplementay data 2.

### Quantitative real-time PCR

Quantitative real-time PCR (qPCR) analysis was performed using total RNA isolated from cells or tissues with Trizol™ reagent (Takara Bio 9109). Subsequently, cDNA was synthesized from 1 μg RNA using the PrimeScript™ RT Reagent Kit (Takara Bio RR047A). qPCR amplification was conducted with TB Green™ Premix Ex Taq™ II (Takara Bio RR820A) on an ABI 7500 Real-Time PCR System, with all reactions performed in triplicate using primers listed in Table S3. Gene expression levels were normalized to ACTB and calculated using the 2^−ΔΔCt^ method.

### Chromatin immunoprecipitation-qPCR (ChIP-qPCR) assay

Chromatin immunoprecipitation (ChIP) was performed using 1 × 10^6^ MCF7 cells crosslinked with 1% formaldehyde (Sigma F8775) for 10 minutes. Subsequently, chromatin was sheared to 200–500 bp fragments using a Covaris M220 (settings: 20% duty cycle, 200 cycles/burst, 180 seconds). The sheared chromatin was then immunoprecipitated overnight at 4 °C with an anti-ERα rabbit monoclonal antibody (Cell Signaling Technology, 8644) pre-bound to Protein G Dynabeads™ (Thermo Fisher 10004D). Following immunoprecipitation, the complexes were washed, eluted, and subjected to crosslink reversal overnight at 65 °C in buffer containing 200 mM NaCl and 10 μg Proteinase K (Thermo Fisher 25530049). Immunoprecipitated DNA was purified using the MinElute PCR Purification Kit (Qiagen 28004). Quantitative PCR was performed in triplicate using TB Green™ Premix Ex Taq™ II (Takara Bio RR820A) on an ABI 7500 Real-Time PCR System. ChIP-qPCR enrichment was calculated as percent input using the formula % Input = 2 ^ (-ΔCt [normalized ChIP IP or IgG]), with background subtraction based on values obtained using rabbit IgG (Cell Signaling Technology 2729). All primers (listed in Table S3) demonstrated amplification efficiencies of 90–110% and included primers for non-targeting genomic regions as negative controls; data represent the mean ± standard deviation (SD) from three independent ChIP experiments, each analyzed with triplicate qPCR reactions.

### Co-immunoprecipitation (co-IP) and mass spectrometry

Co-immunoprecipitation (co-IP) was performed by lysing MCF7 FulR cells on ice in co-IP buffer [50 mM Tris-HCl, pH 7.4, 150 mM NaCl, 1% NP-40, 0.5% sodium deoxycholate, 1× protease inhibitor cocktail (Beyotime P1048)]. Lysates containing 1000 μg total protein were pre-cleared with species-matched rabbit normal IgG (Beyotime A7007) bound to Protein A/G Magnetic Beads (Proteintech HY-K0202, 50% slurry) for 2 hours at 4 °C. The pre-cleared supernatants were then incubated overnight at 4 °C with either anti-ICAM2 rabbit monoclonal antibody (Cell Signaling Technology 13355) or control IgG, both complexed to 25 μL Protein A/G Beads. Following incubation, bead-bound complexes were washed five times with high-salt buffer [20 mM Tris-HCl, pH 7.5, 500 mM NaCl, 0.1% NP-40] and subsequently eluted. For mass spectrometry analysis, the immunoprecipitated complexes underwent additional washes with NP-40 buffer, followed by denaturation in 8 M urea/100 mM Tris (pH 7.6), reduction with 5 mM DTT (37 °C, 30 min), alkylation with 15 mM iodoacetamide (RT, 30 min in dark), and digestion with sequencing-grade trypsin (Promega V5111; 1:50 w/w, 37 °C, 18 h). The resulting peptides were desalted and analyzed using a Q Exactive HF-X mass spectrometer (Thermo Fisher) coupled to a 120-min LC gradient at Metware Technology (Wuhan, China). Data processing was performed using MaxQuant (v2.2.0) against the UniProt human database (2023_07), applying a peptide false discovery rate (FDR) threshold of <1% and requiring a minimum peptide length of 7 amino acids.

### Statistical analysis

In general, the data are presented as the mean standard deviation (SD) from at least three independent experiments. Blinding was not performed. Statistical analysis used GraphPad software; normally distributed data were analyzed with unpaired two-tailed Student’s t-test or paired t-test for two groups. When data are not normally distributed, the non-parametric Mann-Whitney U test was employed. One-way ANOVA was used for multiple comparisons, ensuring similar variance between groups; significance levels were defined as *P < 0.05, **P < 0.01, ***P < 0.001, ****P < 0.0001.

## Supplementary information

supplementary figures and table

Supplementay data 2

A reproducibility checklist

## Data Availability

The data that support the findings of this study are available from the corresponding authors upon reasonable request.
