# Supplementary material for: ICAM2 promotes endocrine resistance via dynein-mediated OXPHOS activation in ER-positive breast cancer
Source: Cell Death Dis. 2026 May 18;17(1):631. doi: 10.1038/s41419-026-08864-1 (PMC13350946; doi:10.1038/s41419-026-08864-1)
Supplement: Supplementary file 1 — supplementary figures and table [file 41419_2026_8864_MOESM1_ESM.pdf]

Supplementary Figures and Legends

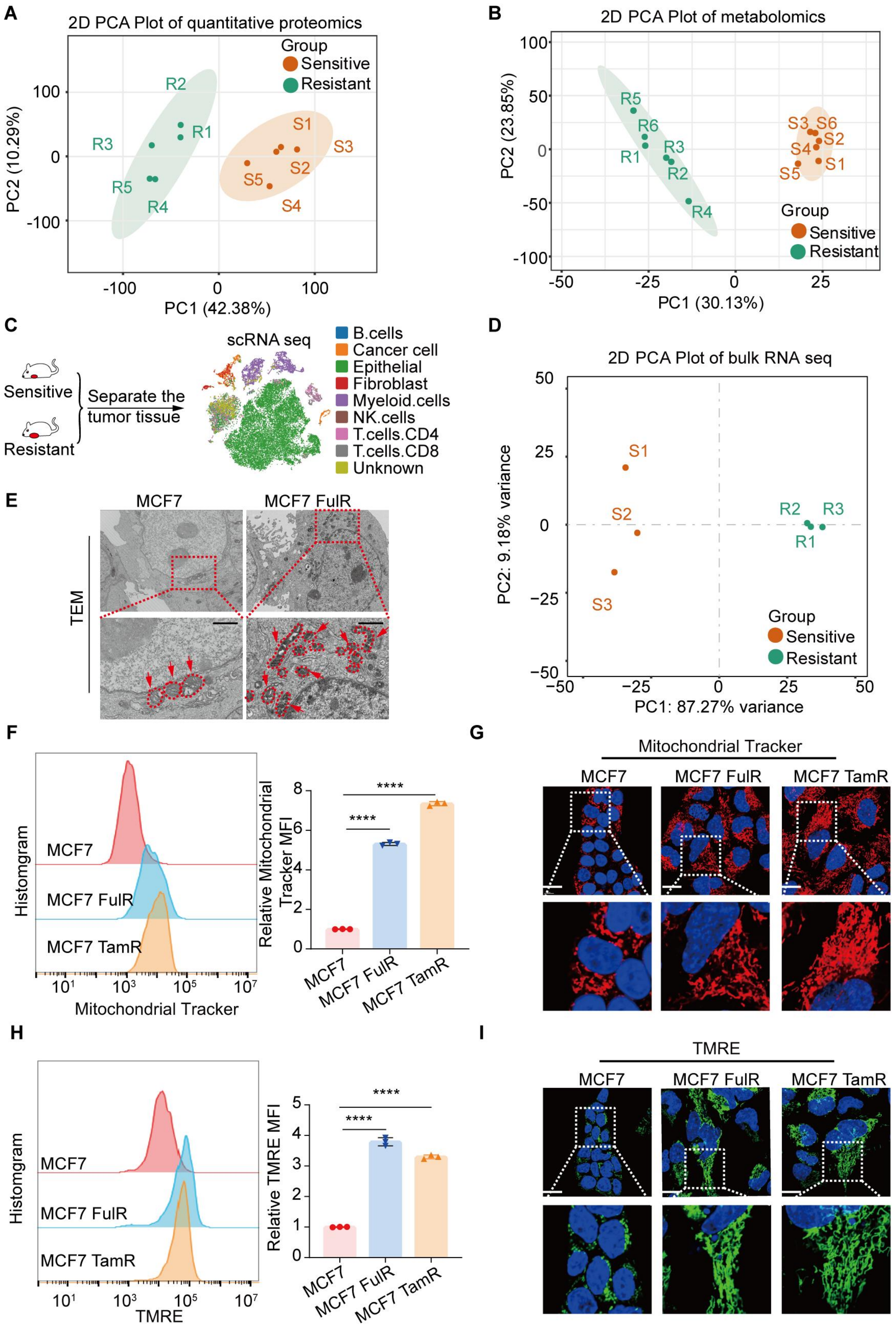

Fig. S1 Multi-omics uncovers enhanced mitochondrial metabolism in endocrine resistance

(A&B) Principal component analysis (PCA) plot of quantitative proteomic profiling (A) and metabolomics (B) of the fulvestrant-sensitive and -resistant mouse model. **(C)** Schematic graph of fulvestrant-resistant ER^+ breast cancer mouse model for scRNA-seq. **(D)**PCA plot of bulk

RNA-seq of cancer cells sorted from the fulvestrant-sensitive and -resistant mouse model. **(E)** TEM was used to visualise mitochondria morphology in MCF7 and MCF7 FulR cells. Scale bars, 10 µm. The red arrowhead: mitochondrion. **(F)** Flow cytometry analysis of MCF7, MCF7 FulR and MCF7 TamR cells. Cells stained with mitochondrial tracker and fluorescence intensities are shown (n = 3, each group). **(G)** Representative mitochondria images depicting MCF7, MCF7 FulR and MCF7 TamR cells stained with Mitochondrial Tracker. Scale bar, 20 µm. **(H)** Flow cytometry anaylzed relative TMRE MFI reflecting mitochondrial membrane potential (TMRE) measured in MCF7, MCF7 FulR and MCF7 TamR cells. n = 3, each group) **(I)**TMRE signals in MCF7, MCF7 FulR and MCF7 TamR cells were detected by fluorescence microscopy. Scale bar, 20 µm. ****$p <$ 0.0001.

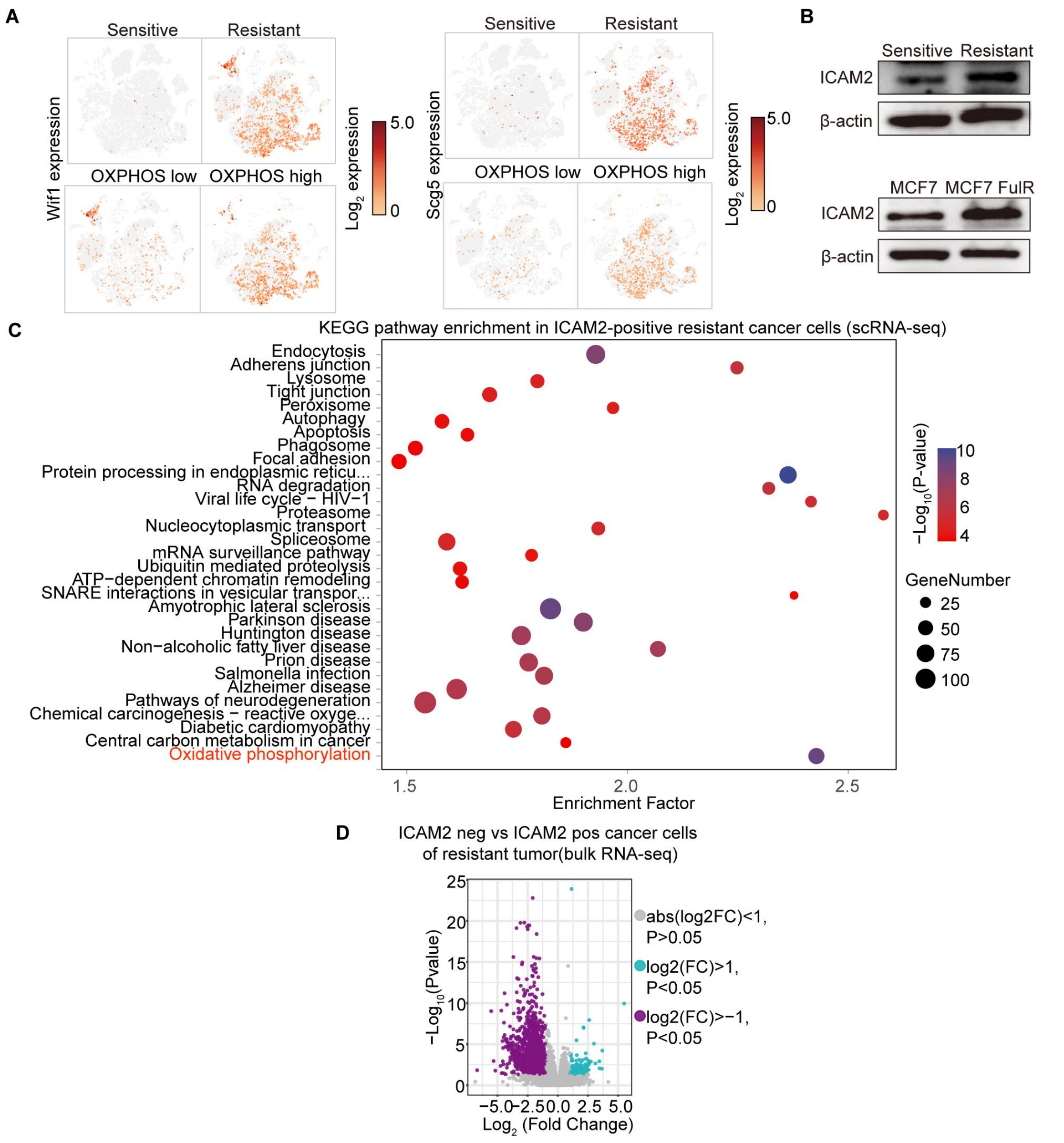

Fig. S2 ICAM2 associated with enhanced OXPHOS in endocrine-resistant cancer

(A)UMAP plot displaying *Wif1* and *Scg5* expression in the fulvestrant-sensitive and -resistant mouse model (up) and within fulvestrant-resistant tumor cells categorized into OXPHOS low and high group (bottom). **(B)**Western Blot analysis of ICAM2 expression in cancer cells ($CD45^-$ $EpCAM^+$) sorted from fulvestrant sensitive or resistant mouse models, MCF7 and MCF7 FulR cells. β-actin was used as loading controls. **(C)** KEGG pathway enrichment analysis was performed in ICAM2 positive resistant cancer cells identified by scRNA-seq.**(D)** Volcano plot depicting differentially expressed genes identified by bulk RNA sequencing of ICAM2 negative and ICAM2 positive cancer cells sorted from tumor tissues of fulvestrant resistant mouse model. $^{**}p < 0.01$, $^{****}p < 0.0001$.

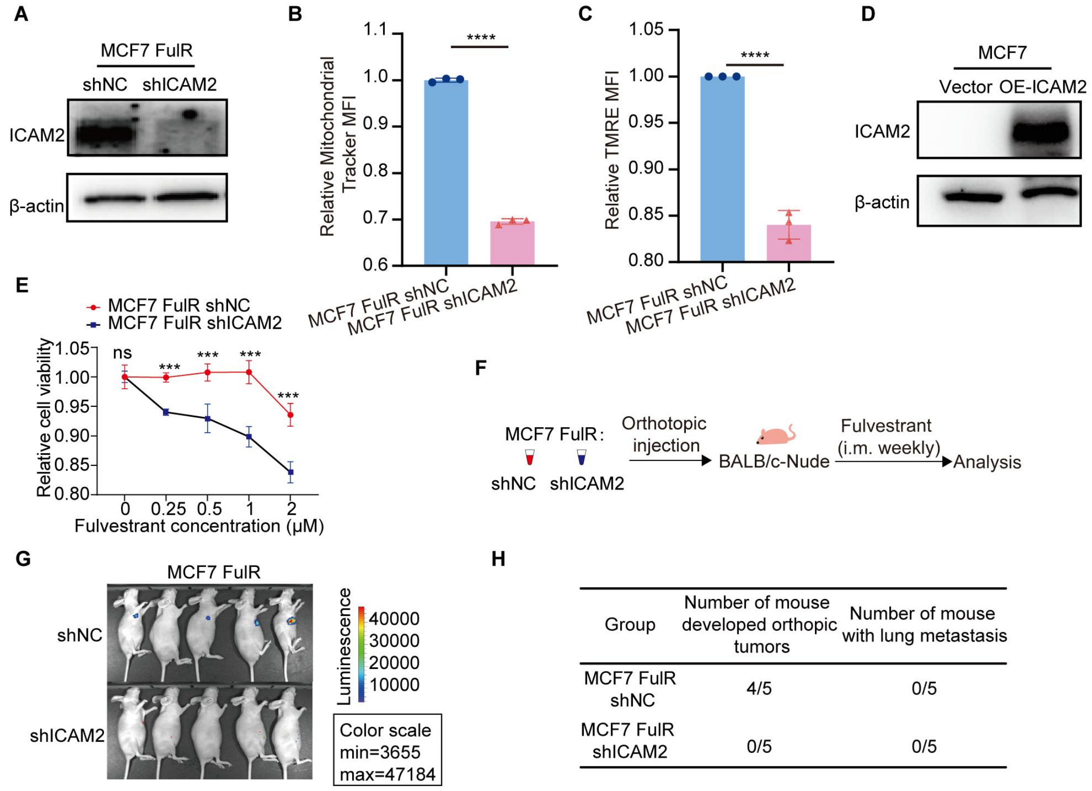

Group	Number of mouse developed orthopic tumors	Number of mouse with lung metastasis
MCF7 FulR shNC	4/5	0/5
MCF7 FulR shICAM2	0/5	0/5

Fig.S3 ICAM2 knockdown reverses fulvestrant resistance in vitro and in vivo.

(A) Western blot analyzed the ICAM2 protein levels in MCF7 FulR cells infected with lentivirus containing control short hairpin(sh) RNA (shNC) or shRNA against ICAM2 (shICAM2). **(B&C)** Flow cytometry analysis of MCF7 FulR shNC/shICAM2 cells by mitochondrial tracker staining(B) and TMRE staining(C). Relative MFI shown (n = 3 biological replicates per group). **(D)** Western

blot analyzed the ICAM2 protein levels in MCF7 cells infected with lentivirus containing control (Vector) or ICAM2-overexpressing plasmid (OE-ICAM2). **(E)** CCK-8 assay assessed fulvestrant sensitivity in MCF7 FulR shNC/shICAM2 cells. **(F)** Schematic diagram of the orthotopic xenograft procedure. MCF7 FulR shNC/shICAM2 cells were orthotopically implanted in female BALB/c nude mice. Weekly fulvestrant treatment (50 mg/kg, i.m.) was given. **(G)** In vivo bioluminescence imaging of tumor development in mouse. **(H)** Orthotopic tumor and metastasis incidence in mice (G).

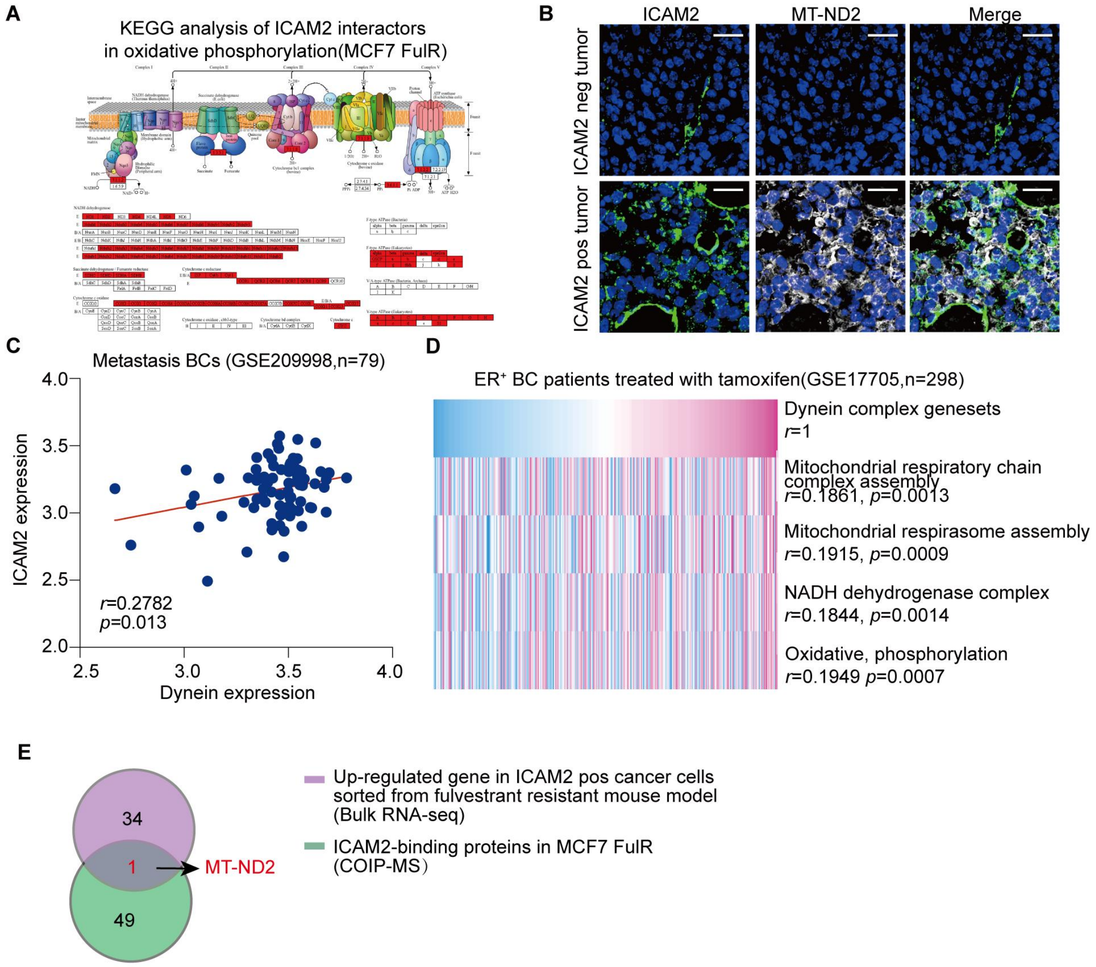

Fig. S4 ICAM2-Dynein-Complex I interaction drives OXPHOS machinery

(A) KEGG pathway map of ICAM2-interacting proteins related with oxidative phosphorylation in MCF7 FulR identified by a co-immunoprecipitation based mass spectrometry assay. **(B)** Representative IF images showing co-localization of ICAM2 (green) and MT-ND2 (white) in orthotopic xenograft tumors derived from ICAM2-negative (ICAM2 neg) or ICAM2-positive (ICAM2 pos) fulvestrant-resistant cancer cells. Scale bar, 10 μm. **(C)** Positive correlation between

ICAM2 and Dynein complex in metastasis breast cancer (BC) patients (GSE209998, n=79). **(D)** Heatmap depicting correlation patterns of the signature in tamoxifen-treated ER$^+$ BCs (GSE17705; n = 298). **(E)** Venn diagram depicting the overlap between genes significantly upregulated in ICAM2-positive cancer cells (identified by Bulk RNA-seq) and proteins identified as direct or indirect interactors of ICAM2 (identified by Co-IP/MS). *$p < 0.05$, **$p < 0.01$, ***$p < 0.001$, ****$p < 0.0001$.

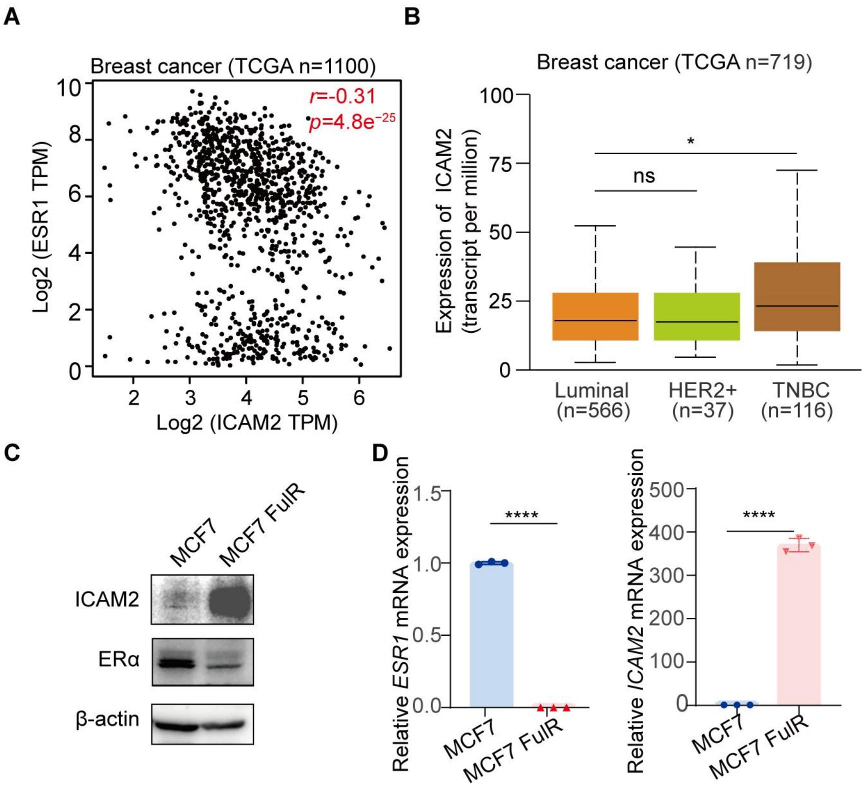

Fig. S5 The negative correlation between ICAM2 and ERα in breast cancer

(A) Negative correlation between ICAM2 and ESR1 in breast cancer patients (TCGA, n=1100). **(B)** The ICAM2 transcript level in human breast cancer datasets of TCGA containing luminal (ER$^+$, n=566), HER2+ (n=37), and triple-negative (TNBC; n=116) subtypes. **(C)** Western blot analyzed ICAM2 and ERα expression levels of MCF7 and MCF7 FulR cells. **(D)** qRT-PCR analysis of ICAM2 and ESR1 mRNA levels in MCF7 and MCF7 FulR cells. Levels were normalized to β-actin. Bars represent mean ± SEM of n = 3 independent samples. ns (no significance). *$p < 0.05$, ****$p < 0.0001$.

Supplementary tables

Table S1 The genesets related to the oxidative phosphorylation pathway

List	Name
Ndufb2	ENSMUSG00000002416
Ndufb6	ENSMUSG00000071014
Ndufc1	ENSMUSG00000037152
Ndufb4	ENSMUSG00000022820
Ndufb3	ENSMUSG00000026032
Ndufa5	ENSMUSG00000023089
Ndufs5	ENSMUSG00000028648
Uqcr10	ENSMUSG00000059534
Ndufa3	ENSMUSG00000035674
Uqcr11	ENSMUSG00000020163
Cox7b	ENSMUSG00000031231
Cox7a2	ENSMUSG00000032330
Cox7c	ENSMUSG00000017778
Cox8a	ENSMUSG00000035885
Cycs	ENSMUSG00000063694
Park7	ENSMUSG00000028964
Ndufa7	ENSMUSG00000041881
Ndufb8	ENSMUSG00000025204
Uqcrq	ENSMUSG00000044894
Uqcrb	ENSMUSG00000021520
Ndufb11	ENSMUSG00000031059
Ndufab1	ENSMUSG00000030869
Ndufb5	ENSMUSG00000027673
Ndufs6	ENSMUSG00000021606
Ndufa13	ENSMUSG00000036199
Ndufc2	ENSMUSG00000030647
Cox5a	ENSMUSG00000000088
Cox5b	ENSMUSG00000061518
Ndufa2	ENSMUSG00000014294
Iscu	ENSMUSG00000025825
Ndufb7	ENSMUSG00000033938
Chchd10	ENSMUSG00000049422
Dnajc15	ENSMUSG00000022013
Uqcc2	ENSMUSG00000024208
Ndufs7	ENSMUSG00000020153
Uqcrh	ENSMUSG00000063882
Uqcrc2	ENSMUSG00000030884
Ndufa8	ENSMUSG00000026895
Ndufs8	ENSMUSG00000059734
Ndufv2	ENSMUSG00000024099
Ndufa9	ENSMUSG00000000399

Ndufa11	ENSMUSG00000002379
Ndufv3	ENSMUSG00000024038
Ndufa12	ENSMUSG00000020022
Ndufs2	ENSMUSG00000013593
Uqcrfs1	ENSMUSG00000038462
Ndufa10	ENSMUSG00000026260
Ndufb10	ENSMUSG00000040048
Sdhb	ENSMUSG00000009863
Cyc1	ENSMUSG00000022551
Ndufs3	ENSMUSG00000005510
Uqcc3	ENSMUSG00000071654
Uqcrc1	ENSMUSG00000025651
Cox6a1	ENSMUSG00000041697
Ndufb9	ENSMUSG00000022354
Chchd2	ENSMUSG00000070493
Ndufs4	ENSMUSG00000021764
Ndufa6	ENSMUSG00000022450
Ndufv1	ENSMUSG00000037916
Stoml2	ENSMUSG00000028455
Cox7a1	ENSMUSG00000074218
Mtch2	ENSMUSG00000027282
Cox7a2l	ENSMUSG00000024248
Sdhc	ENSMUSG00000058076
Cox4i1	ENSMUSG00000031818
Bid	ENSMUSG00000004446
Atpsckmt	ENSMUSG00000039065
Snca	ENSMUSG00000025889
Ndufs1	ENSMUSG00000025968
Dguok	ENSMUSG00000014554
Dld	ENSMUSG00000020664
Ndufaf1	ENSMUSG00000027305
Coq7	ENSMUSG00000030652
Nipsnap2	ENSMUSG00000029432
Coq9	ENSMUSG00000031782
Coa6	ENSMUSG00000051671
Dnajc30	ENSMUSG00000061118
Fxn	ENSMUSG00000059363
Sdhaf2	ENSMUSG00000024668
Pink1	ENSMUSG00000028756
Slc25a23	ENSMUSG00000046329
Tefm	ENSMUSG00000046909
Afg1l	ENSMUSG00000038302
Pde2a	ENSMUSG00000110195
Cox8b	ENSMUSG00000025488

Table S2. Clinicopathological characteristics in the studied cohort, related to Figure 7&8.

Tissue marker		N	ICAM2 intensity of area(%) Median (25% Percentile, 75% Percentile)	*P* value
Age	<50	45	2.420(1.025, 4.855)	0.7889
	≥50	80	2.095(1.368, 4.475)	
Tumor stage	T1	47	2.260(1.130, 4.330)	0.8477
	T2	76	2.150(1.175, 5.668)	
	T3	2	3.045(2.680, 3.410)	
Nodal stage	N0	62	2.125(1.103, 4.305)	0.0764
	N1	18	1.530(0.900, 2.450)	
	N2-3	45	3.350(1.415, 6.310)	
Tumor grade	II	85	1.860(1.025, 3.995)	0.5403
	II-III	36	3.430(1.670, 6.130)	
	III	5	2.420(2.005, 3.270)	
Relapse	non-relapse	89	2.070(1.020, 4.570)	0.0228
	relapse	36	2.910(1.648, 5.125)	
ER^+		61	1.560(0.940, 3.560)	/
HER2		38	1.860(1.190, 3.100)	0.7141
TNBC		26	3.395(1.713, 4.258)	0.0307

Difference between groups was tested by Mann-Whitney test or Kruskal Wallis test.

Table S3. Key resource table

Antibodies and reagents

Reagents	Source	Identifier
anti-human/mouse ICAM2	Cell Signaling Technology	13355
anti-mouse ICAM2	Abcam	ab189463
FITC anti-mouse CD45	Invitrogen	11-0451
PE anti-mouse EpCAM	Invitrogen	12-5791
anti-human/mouse β-actin	Servicebio	GB15001-100
anti-human Dynein	Proteintech	11687-1-AP
anti-human/mouse MT-ND2	Thermo Fisher	PA5-103952
PE anti-human CD102 (ICAM-2)	BioLegend	328505
PE anti-human CD102 (ICAM-2)	Thermo Fisher	A15451
anti-human ERα	Abcam	ab64693
Alexa Fluor® 488-conjugated goat anti-rabbit IgG	Thermo Fisher	A-11008
Alexa Fluor® 594-conjugated donkey anti-Rabbit IgG	Thermo Fisher	A-11012
Alexa Fluor® 647-conjugated donkey	Thermo Fisher	A-31573

anti-Rabbit IgG		
Anti-Rabbit IgG, HRP-linked Antibody	Cell Signaling Technology	7074
Anti-Mouse IgG, HRP-linked Antibody	Cell Signaling Technology	7076
Alexa Fluor® 488-conjugated goat anti-mouse IgG	Thermo Fisher	A-11001
Alexa Fluor® 594-conjugated goat anti-mouse IgG	Thermo Fisher	A-11005
Alexa Fluor® 647-conjugated goat anti-mouse IgG	Thermo Fisher	A-31571
Fulvestrant	Selleck	S1191
Liberase TL	Roche	05401020001
DNAse I	Roche	11284932001
CCK-8	NCM biotech	C6005
IACS-10759	MCE	HY-112037
Ciliobrevin D	MCE	HY-122632

Deposited data

Raw data files for RNA-seq	Li A et al., 2019;<br>Symmans WF et al., 2010;<br>Pawitan Y et al.,2005;<br>Kelly BJ et al. , 2023;<br>Booser DJ et al., 2011.	GEO:GSE111563;<br>GEO:GSE17705;<br>GEO:GSE1456;<br>GEO:GSE209998;<br>GEO:GSE25066.
TCGA datasets	TIMER2.0	

Software and algorithms

FlowJo v10Tree Star	FlowJo	https://www.flowjo.com/
R 4.4.3	R	https://www.r-project.org/
Image J 1.53.c	Image J	https://imagej.net/software/ima Image J 1.53.c Image.J gej
GraphPad Prism10	GraphPad	https://www.graphpad.com/

Primer list

Site		Sequences
ACTB	Forward	5'-CACCATTGGCAATGAGCGGTTC-3'
	Reverse	5'-AGGTCTTTGCGGATGTCCACGT-3'
ESR1	Forward	5'-GCTTACTGACCAACCTGGCAGA-3'
	Reverse	5'-GGATCTCTAGCCAGGCACATTC-3'
ICAM2	Forward	5'-ATGACACGGTCCTCCAATGCCA-3'
	Reverse	5'-GCACTCAATGGTGAAGGACTTGC-3'
MT-ND1	Forward	5'-CGCACTCTCCCCTGAACTCT-3'
	Reverse	5'-AGTTGGTCGTAGCGGAATCG-3'
MT-ND2	Forward	5'-CCCGCTAACCGGCTTTTT-3'

	Reverse	5'-GGAGGGTGATGGTGGCTATG-3'
MT-ND3	Forward	5'-TGCGGCTTCGACCCTATATC-3'
	Reverse	5'-AGGGGTAAAAGGAGGGCAAT-3'
MT-ND4	Forward	5'-CTTTTCCTCCGACCCCCTAA-3'
	Reverse	5'-GGATAAGTGGCGTTGGCTTG-3'
MT-CYB	Forward	5'-AGACAGTCCCACCCTCACAC-3'
	Reverse	5'-GTTGTTTGATCCCGTTTCGT-3'
MT-CO1	Forward	5'-AGGGGCCATCAATTTCATCA-3'
	Reverse	5'-GGACGGATCAGACGAAGAGG-3'
MT-CO2	Forward	5'-GATTGAAGCCCCCATTCGTA-3'
	Reverse	5'-TCCGGGAATTGCATCTGTTT-3'
MT-CO3	Forward	5'-TGTCCCAGATGAACGTCACAGC-3'
	Reverse	5'-TGGTGGCTGTTTCACTGGAGCA-3'
ATP6	Forward	5'-GCGGGCGCAGTGATTATAGG-3'
	Reverse	5'-ATGGGGATAAGGGGTGTAGG-3'
ATP8	Forward	5'-AACATTCCCACTGGCACCTTC-3'
	Reverse	5'-TGGGGTAATGAATGAGGCAAATAG-3'

Primers for ChIP-qPCR

Site		Sequences
a	Forward	5'-GGAGAATTGCTTGAACTCGGG-3'
	Reverse	5'-TGTTTTGTAGAGATGGAGCCTC-3'
b	Forward	5'-CAGAGGTTACAGTGAGCCAAGA-3'
	Reverse	5'-AGCTCTCTCTTTCTCTTTTCTTTCTTT-3'
c	Forward	5'-GAAGTTGAGGAGGGAGGGTC-3'
	Reverse	5'-AGAGTCTTGCTTTTGTCACCT-3'
