# Supplementary material for: ICAM2 promotes endocrine resistance via dynein-mediated OXPHOS activation in ER-positive breast cancer
Source: Cell Death Dis. 2026 May 18;17(1):631. doi: 10.1038/s41419-026-08864-1 (PMC13350946; doi:10.1038/s41419-026-08864-1)
Supplement: Supplementary file 2 — Supplementay data 2 [file 41419_2026_8864_MOESM2_ESM.pdf]

Fig5C

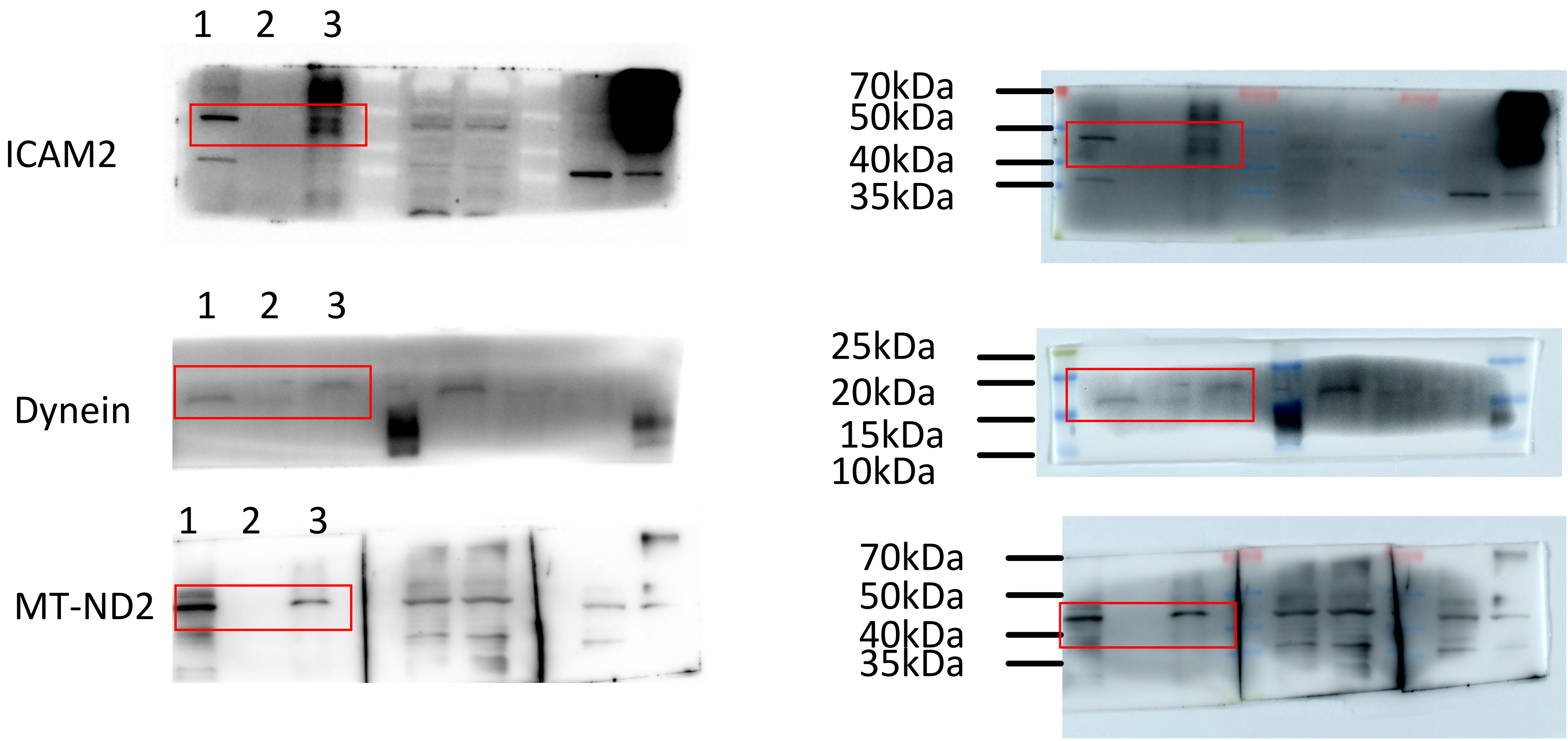

1 MCF7 FulR Input
2 MCF7 FulR IgG
3 MCF7 FulR ICAM2 IP

Fig.7A

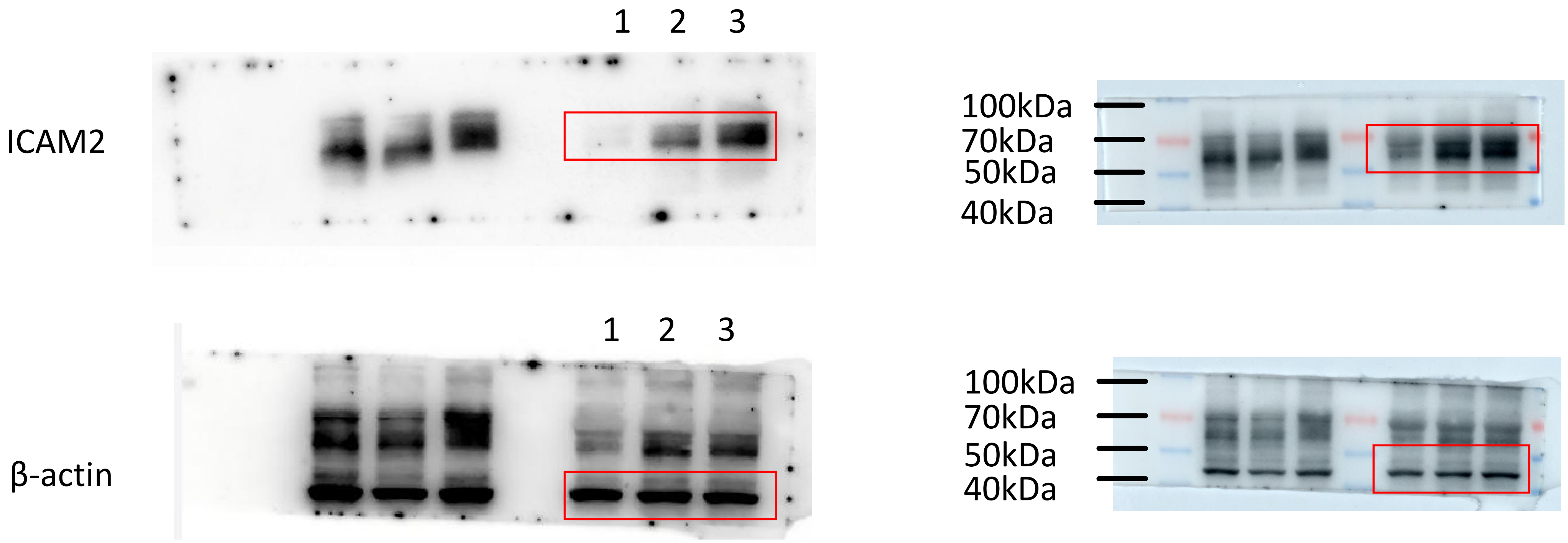

1 MCF7 vehicle
2 MCF7 Tam
3 MCF7 Ful

Fig.7E

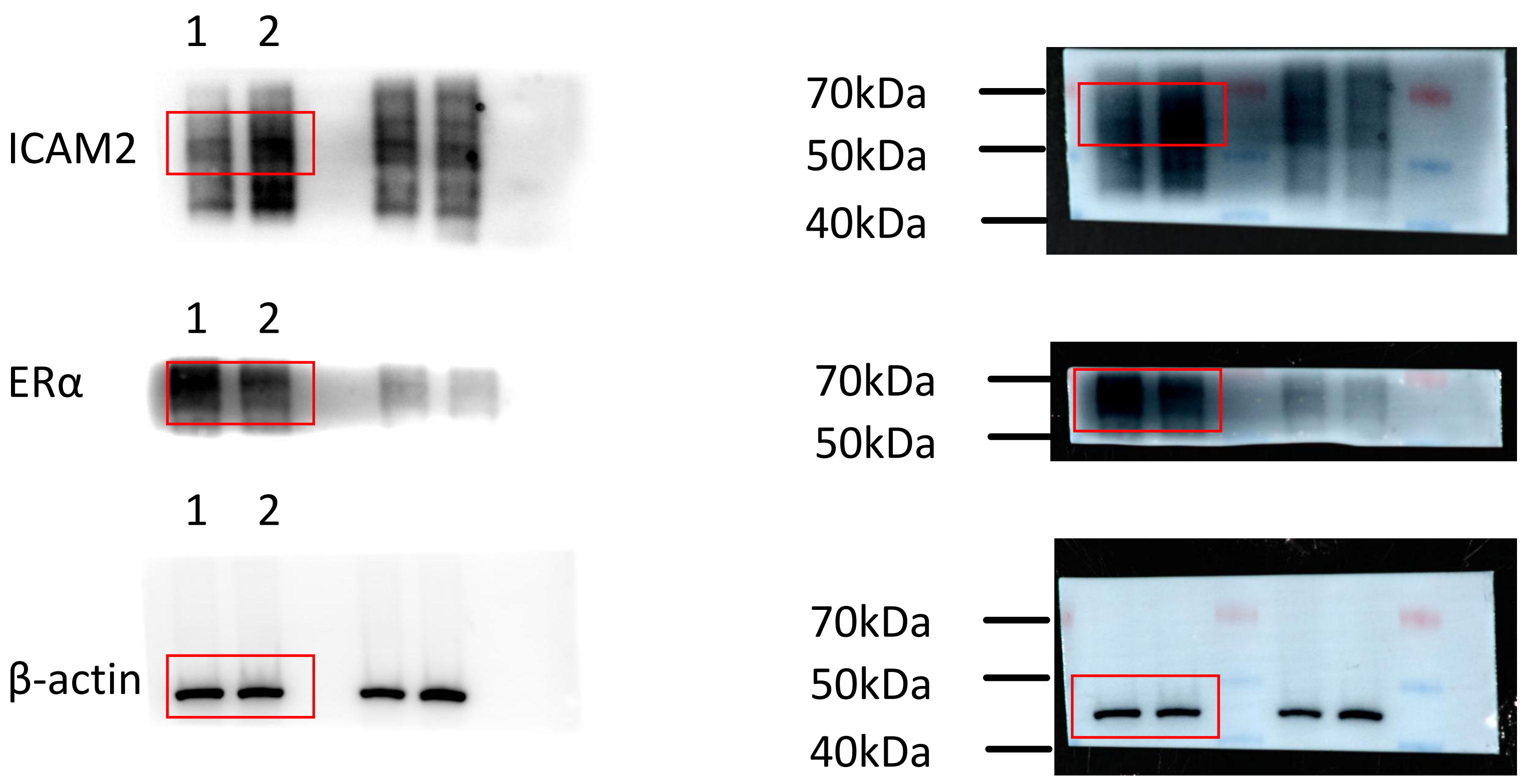

1 MCF7 siNC
2 MCF7 siESR1

Fig S2B

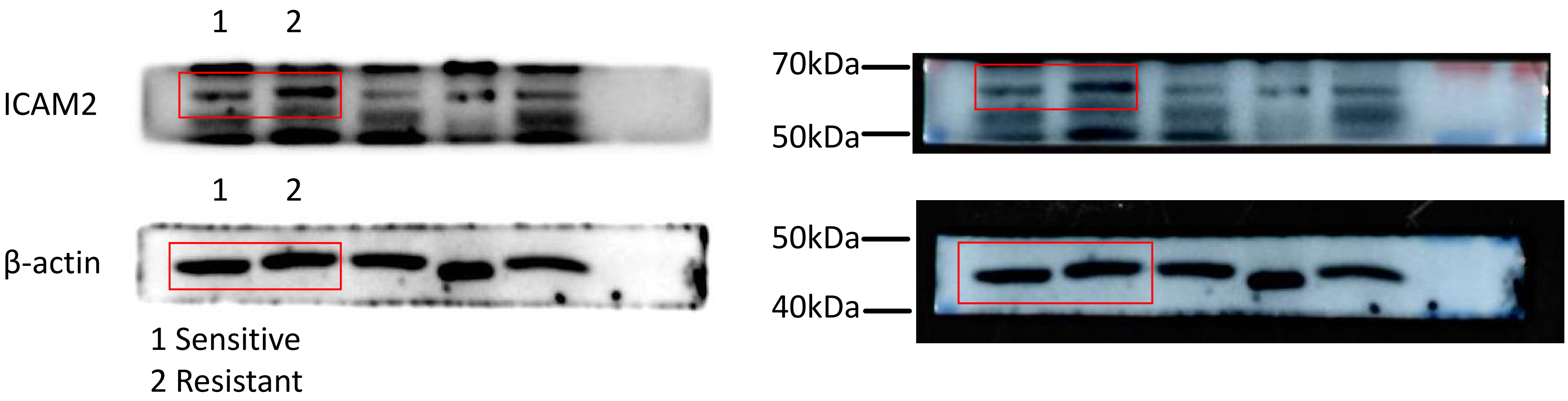
1
2
ICAM2
1
2
β-actin
1 Sensitive
2 Resistant
70kDa
50kDa
50kDa
40kDa

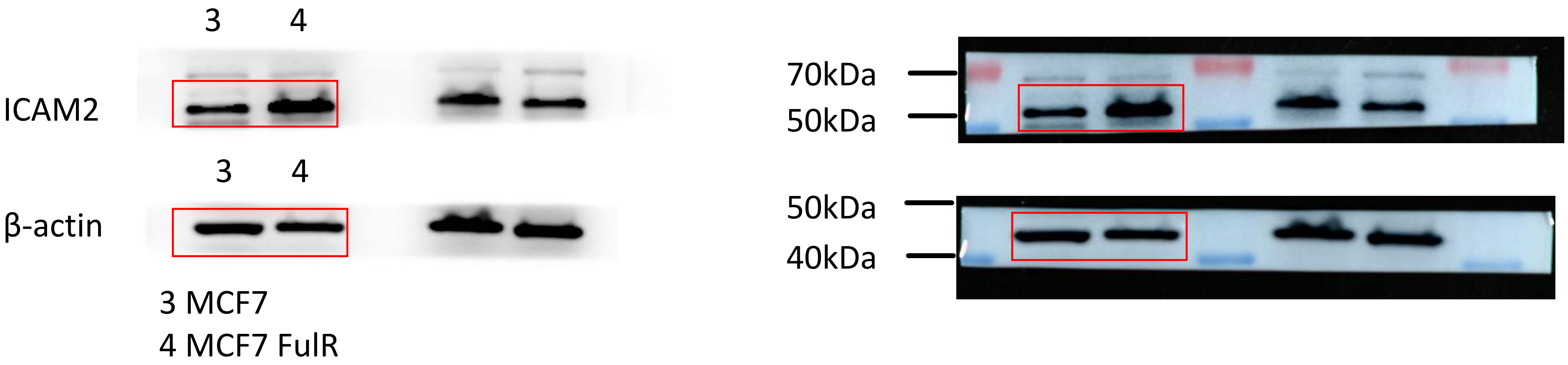
3
4
ICAM2
3
4
β-actin
3 MCF7
4 MCF7 FulR
70kDa
50kDa
50kDa
40kDa

Fig S3A

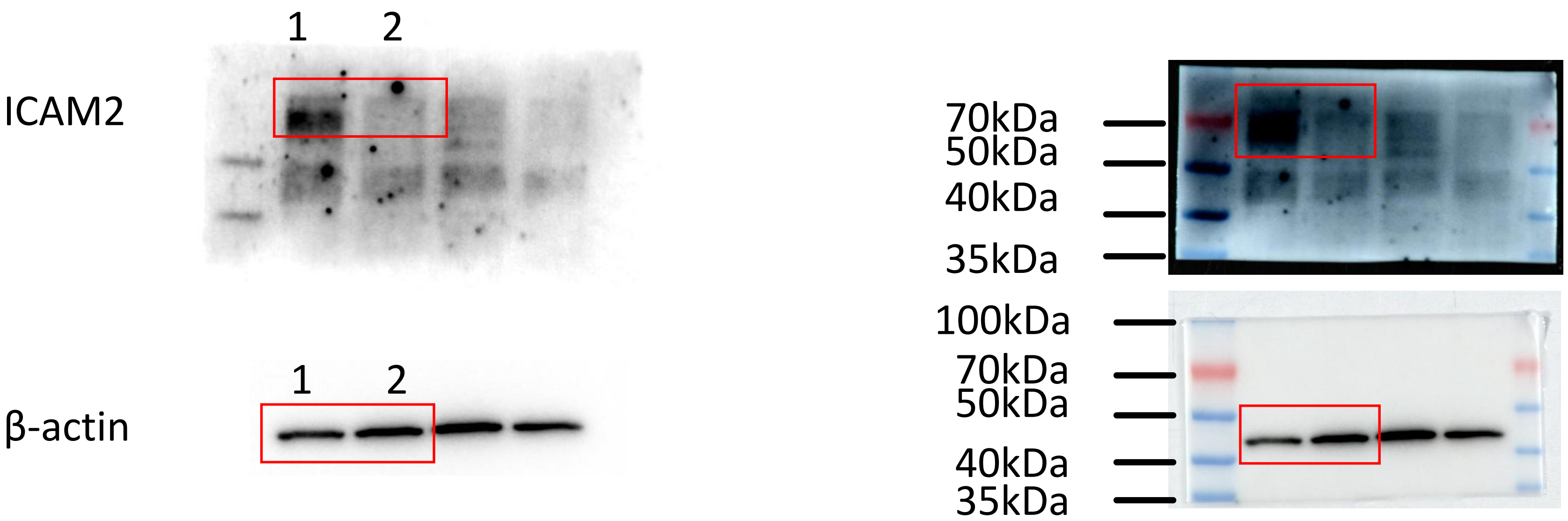

1 MCF7 FulR shNC
2 MCF7 FulR shICAM2

Fig S3D

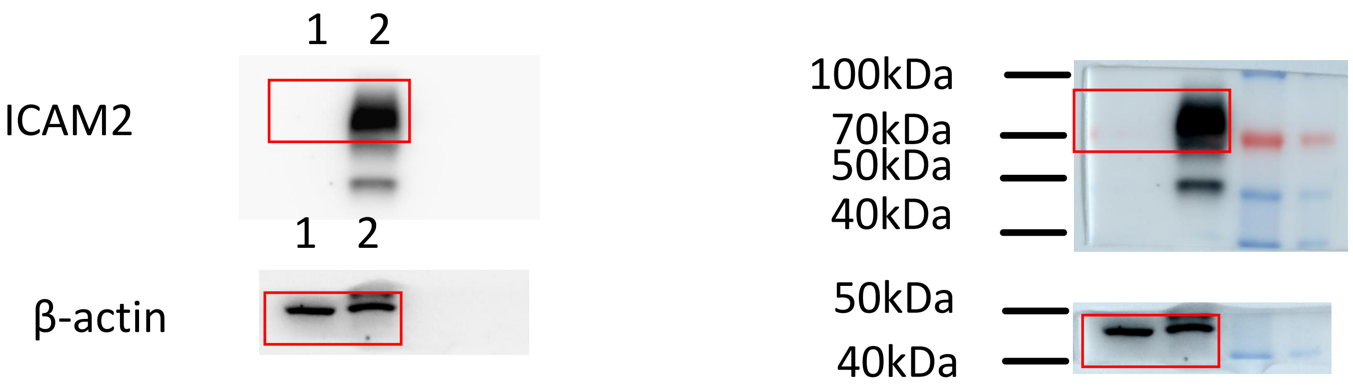

1 MC7 Vector
2 MCF7 OE-ICAM2

Fig S5C

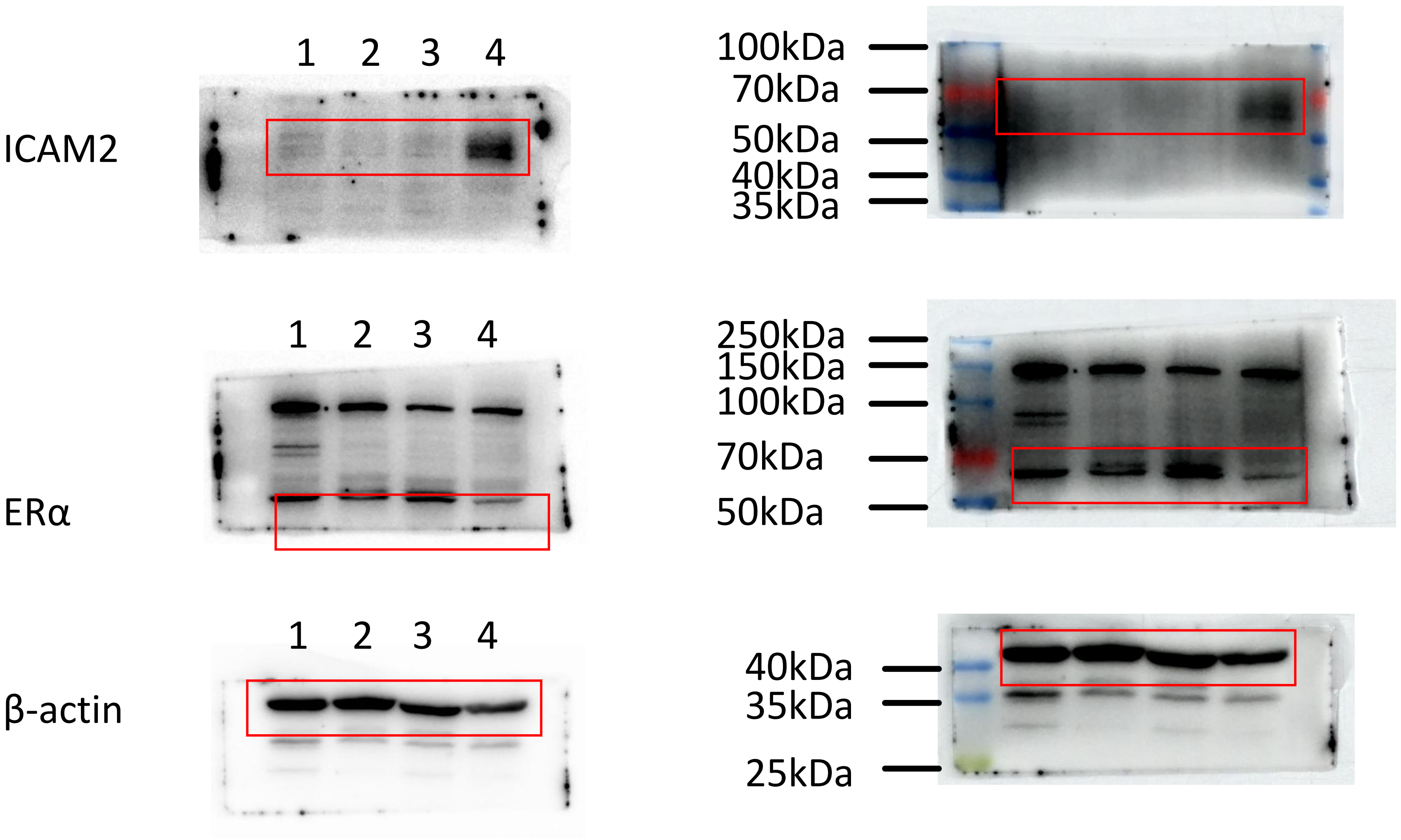

1 E0771
2 T47D
3 MCF7
4 MCF7 FulR
